# Supplementary material for: High-energy resolution X-ray spectroscopy reveals bonding characteristics of La3+ homologues of actinium radiopharmaceuticals
Source: Commun Chem. 2026 Mar 11;9:148. doi: 10.1038/s42004-026-01929-4 (PMC13065765; doi:10.1038/s42004-026-01929-4)
Supplement: Supplementary file 2 — Supplementary Information [file 42004_2026_1929_MOESM2_ESM.pdf]

High-energy resolution X-ray spectroscopy reveals bonding characteristics of La^{3+} homologues of actinium radiopharmaceuticals

Harry Ramanantoanina[1], Bianca Schacherl[1], Attila Kovács[2], Michelangelo Tagliavini[3], Emily Marie Reynolds[1], Cedric Reitz[1], Ruwini S. K. Ekanayake[1], Martin Schäfer[4], Paul-Valentin von Massow[1], Jörg Göttlicher[5], Ralph Steininger[5], Kathy Dardenne[1], Maurits W. Haverkort[3], Martina Benešová-Schäfer[6], and Tonya Vitova[1]

[1]Karlsruhe Institute of Technology (KIT), Institute for Nuclear Waste Disposal (INE), P.O. Box 3640, D-76021 Karlsruhe, Germany
[2]Joint Research Centre (JRC), European Commission, DE-76125 Karlsruhe, Germany
[3]German Cancer Research Center (DKFZ), Service Unit for Radiopharmaceuticals and Preclinical Trials, DE-69120 Heidelberg, Germany
[4]Karslruher Instituet of Technology (KIT), Institute for Photon Science and Synchrotron Radiation (IPS), DE-76021 Karlsruhe, Germany
[5]University of Heidelberg, Institute for Theoretical Physics, DE-69120 Heidelberg, Germany
[6]German Cancer Research Center (DKFZ), Translational Theranostics, DE-69120 Heidelberg, Germany

January 12, 2026

SUPPLEMENTARY INFORMATION (SI)

Contents

1 Supplementary Experimental Method **2**
1.1 Chemistry, Synthesis and Quality Control . . . 2
1.2 Samples Preparation . . . 2
1.3 Additional EXAFS information . . . 2

2 Supplementary Theoretical Method **3**

3 Additional Supplementary Data **5**

Supplementary References **37**

1 Supplementary Experimental Method

1.1 Chemistry, Synthesis and Quality Control

All chemicals (>95% pure) and solvents (HPLC-grade) were purchased from Bachem, Carbolution, CheMatech, Fluka, Iris Biotech, Macrocyclics, Merck Group, Carl Roth or Sigma Aldrich and directly used without any further re-processing. *TRIS* (tris(hydroxymethyl)aminomethane) was purchased from Sigma Aldrich and *DOTA* chelator ($2,2',2'',2'''$-(1,4,7,10-Tetra azacyclododecane-1,4,7,10-tetrayl) tetraacetic acid) from CheMatech. *MACROPA* chelator and *PSMA*-617 were synthesized as reported in refs. [1, 2] purified by semi-preparative high-performance liquid chromatography (HPLC), and analyzed by analytical HPLC, electrospray ionization mass spectrometry (ESI-MS) and/or matrix-assisted laser desorption/ionization MS (MALDI-MS). Sample preparations and method are available in the Supplementary Information.

1.2 Samples Preparation

9H2O 196 μL 0.05 M HNO_3 solution was spiked with 4 μL of our 1 M La^{3+} stock solution

TRIS 36 μL of 0.05M HNO_3 was mixed with 4 μL 1M La^{3+} stock solution, and 140μL 0.06M *TRIS* solution at pH = 9.

MACROPA In a 1.5 mL low-bind Eppendorf tube, a mixture of 36 μL 0.05M HNO_3, 40 μL 1M La^{3+} stock, 120 μL 0.625 M *TRIS* at pH = 9.0, and 40 μL 100mM *MACROPA* solution were reacted at room temperature.

DOTA In a 1.5 mL low-bind Eppendorf tube, a mixture of 36 μL 0.05M HNO_3, 4 μL 1M La^{3+} stock, 120 μL 0.625M *TRIS* at pH 9.0, and 40 μL 100mM *DOTA* solution were heated at 95°C for 30 min.

PSMA In a 1.5 mL low-bind Eppendorf tube, a mixture of 36 μL 0.05M HNO_3, 4 μL 1M La^{3+} stock, 120 μL 0.625M *TRIS* at pH 9.0, and 40 μL 100mM *PSMA*-617 solution were heated at 95°C for 30 min.

All sample solution were filled inside peek cells for measurements on liquid samples described elsewhere and sealed using O-rings and 13μm thick Kapton foils.[3]

1.3 Additional EXAFS information

9H2O It was assumed that a shell of water molecules surrounded the La^{3+} ion. Therefore, a single scattering path of La-O at 2.55 Å was calculated and used as a basis for the fit. The number of variables was 4. The number of independent data points was 5.5. The achieved goodness of fit parameter was r = 0.015, representing a 1.5% difference between the data and the model.

TRIS A similar approach to the 9H2O spectrum was used. The number of variables was 4. The number of independent data points was 6. The achieved goodness of fit parameter was $r = 0.017$, representing a 1.7% difference between the data and the model. Note thta it could not be ruled out that the TRIS ligand forms a complex with La^{3+}.

MACROPA We could not perfom data analysis on the MACROPA measurements due to very low signal to noise ratio.

DOTA For the spectra showing multiple shells in R a simulation of the spectra was performed, therefore the single scattering paths were generated with the Feff6 code[4] using the DFT optimized structures (see below). The coordination numbers were fixed to $N_O = 5$; $N_N = 4$ and $N_C = 16$ to match the model. Only one σ^2 was used as an approximation for all shells to decrease the number of needed parameters. The number of variables was 5. The number of independent data points was 7.92. The achieved goodness of fit parameter was $r = 0.0017$, representing a 1.7% difference between the data and the model.

PSMA Because of the similarity between PSMA and DOTA a similar model was used as a start for the simulation. The number of variables was 5. The number of independent data points was 7.87. The achieved goodness of fit parameter was $r = 0.0014$, representing a 1.4% difference between the data and the model.

2 Supplementary Theoretical Method

The Kramers-Heisenberg formula where used to simulate RIXS processes with respectively ingoing (outgoing) polarization $\vec{\epsilon}_{in(out)}$, photon momentum $\hbar\vec{k}_{in(out)}$ and energy $\omega_{in(out)}$:

$$I(\vec{\epsilon}_{in}, \vec{\epsilon}_{in}, \vec{k}_{in}, \vec{k}_{out}, \omega_{in}, \omega_{out}) \propto \sum_f \Big| \sum_m \langle f| (\hat{T}_{\vec{k}_{out},\vec{\epsilon}_{out}})^\dagger \frac{|m\rangle\langle m|}{\omega_{in} - E_m + E_g + i\Gamma_m} \hat{T}_{\vec{k}_{in},\vec{\epsilon}_{in}} |g\rangle \Big|^2$$
$$\delta(\omega_{in} - \omega_{out} - E_g + E_f). \quad \text{(S1)}$$

where, $\hat{T}_{\vec{k},\vec{\epsilon}}$ indicates the transition operators for photon-matter interaction. For electric-dipole transitions,

$$\hat{T}^{E1}_{\vec{k},\vec{\epsilon}} \propto \sum_{q=-1}^{1} \underbrace{\epsilon^{(1)}_m}_{\tau^1_m} \hat{C}^{(1)}_m, \quad \text{(S2)}$$

and electric-quadrupoles transitions,

$$\hat{T}^{E2}_{\vec{k},\vec{\epsilon}} \propto \sum_{q=-1}^{1} \underbrace{\{k^{(1)}, \epsilon^{(1)}\}^{(2)}_m}_{\tau^2_m} \hat{C}^{(2)}_m. \quad \text{(S3)}$$

Here we choose to expand the operators on a spherical tensor operator basis and the coupling of two spherical tensors is given as:

$$\{A^{(l_1)}_{m_1} \otimes B^{(l_2)}_{m_2}\}^{(l)}_m = \sum_{m_1,m_2} \langle l_1, m_1; l_2, m_2 | l, m\rangle A^{(l_1)}_{m_1} B^{(l_2)}_{m_2}. \quad \text{(S4)}$$

We use the coefficients τ^l_m to introduce a compact labeling of the components in:

$$I(\tau^{l'}_{m'}, \tau^l_m) \propto \sum_f \Big| \sum_m \langle f| (\tau^{l'}_{m'} \hat{C}^{(l')}_{m'})^\dagger \frac{|m\rangle\langle m|}{\omega_{in} - E_m + E_g + i\Gamma_m} (\tau^l_m \hat{C}^{(l)}_m) |g\rangle \Big|^2$$
$$\delta(\omega_{in} - \omega_{out} - E_g + E_f). \quad \text{(S5)}$$

For a 172° σ geometry and considering filtering due to the Sul-X Bragg Spectrometer[5] the spectra is given by:

$$\begin{aligned}
I_{Sul-X} =& 0.320185 I\left((\tau^1{}_{-1})_{\text{In}}, (\tau^1{}_0)_{\text{Out}}\right) + 0.320185 I\left((\tau^1{}_{-1})_{\text{In}}, (\tau^1{}_{-1})_{\text{Out}}\right) + 0.320185 I\left((\tau^1{}_{-1})_{\text{In}}, (\tau^1{}_1)_{\text{Out}}\right) \\
&+ 0.026881 I\left((\tau^2{}_0)_{\text{In}}, (\tau^1{}_0)_{\text{Out}}\right) + 0.0220029 I\left((\tau^2{}_0)_{\text{In}}, (\tau^1{}_{-1})_{\text{Out}}\right) + 0.0220029 I\left((\tau^2{}_0)_{\text{In}}, (\tau^1{}_1)_{\text{Out}}\right) \\
&+ 0.0125844 I\left((\tau^2{}_0)_{\text{In}}, (\tau^1{}_{+1} + \text{i}\tau^1{}_{-1})_{\text{Out}}\right) + 0.0125844 I\left((\tau^2{}_0)_{\text{In}}, (\tau^1{}_{+1} + \tau^1{}_{-1})_{\text{Out}}\right) \\
&+ 0.00444926 I\left((\tau^2{}_0 + \text{i}\tau^2{}_1)_{\text{In}}, (\tau^1{}_0 + \text{i}\tau^1{}_{+1})_{\text{Out}}\right) + 0.00222463 I\left((\tau^2{}_0 + \text{i}\tau^2{}_1)_{\text{In}}, (\tau^1{}_{-1})_{\text{Out}}\right) \\
&- 0.00222463 I\left((\tau^2{}_0 + \text{i}\tau^2{}_1)_{\text{In}}, (\tau^1{}_1)_{\text{Out}}\right) - 0.00444926 I\left((\tau^2{}_0 + \text{i}\tau^2{}_1)_{\text{In}}, (\tau^1{}_{-1} + \text{i}\tau^1{}_0)_{\text{Out}}\right) \\
&+ 0.00629221 I\left((\tau^2{}_0 + \text{i}\tau^2{}_2)_{\text{In}}, (\tau^1{}_{-1})_{\text{Out}}\right) + 0.00629221 I\left((\tau^2{}_0 + \text{i}\tau^2{}_2)_{\text{In}}, (\tau^1{}_1)_{\text{Out}}\right) \\
&- 0.0125844 I\left((\tau^2{}_0 + \text{i}\tau^2{}_2)_{\text{In}}, (\tau^1{}_{+1} + \text{i}\tau^1{}_{-1})_{\text{Out}}\right) - 0.00444926 I\left((\tau^2{}_0 + \tau^2{}_1)_{\text{In}}, (\tau^1{}_0 + \tau^1{}_{+1})_{\text{Out}}\right) \\
&- 0.00222463 I\left((\tau^2{}_0 + \tau^2{}_1)_{\text{In}}, (\tau^1{}_{-1})_{\text{Out}}\right) + 0.00222463 I\left((\tau^2{}_0 + \tau^2{}_1)_{\text{In}}, (\tau^1{}_1)_{\text{Out}}\right) \\
&+ 0.00444926 I\left((\tau^2{}_0 + \tau^2{}_1)_{\text{In}}, (\tau^1{}_{-1} + \tau^1{}_0)_{\text{Out}}\right) + 0.00629221 I\left((\tau^2{}_0 + \tau^2{}_2)_{\text{In}}, (\tau^1{}_{-1})_{\text{Out}}\right) \\
&+ 0.00629221 I\left((\tau^2{}_0 + \tau^2{}_2)_{\text{In}}, (\tau^1{}_1)_{\text{Out}}\right) - 0.0125844 I\left((\tau^2{}_0 + \tau^2{}_2)_{\text{In}}, (\tau^1{}_{+1} + \tau^1{}_{-1})_{\text{Out}}\right) \\
&+ 0.0294497 I\left((\tau^2{}_{-1})_{\text{In}}, (\tau^1{}_0)_{\text{Out}}\right) + 0.0294497 I\left((\tau^2{}_1)_{\text{In}}, (\tau^1{}_0)_{\text{Out}}\right) \\
&+ 0.00767384 I\left((\tau^2{}_{-1})_{\text{In}}, (\tau^1{}_0 + \text{i}\tau^1{}_{+1})_{\text{Out}}\right) - 0.00767384 I\left((\tau^2{}_1)_{\text{In}}, (\tau^1{}_0 + \text{i}\tau^1{}_{+1})_{\text{Out}}\right)
\end{aligned}$$

$$
\begin{aligned}
&- 0.00767384I\left((\tau^2{}_{-1})_{\mathrm{In}}, (\tau^1{}_0 + \tau^1{}_{+1})_{\mathrm{Out}}\right) + 0.00767384I\left((\tau^2{}_1)_{\mathrm{In}}, (\tau^1{}_0 + \tau^1{}_{+1})_{\mathrm{Out}}\right)\\
&+ 0.0255966I\left((\tau^2{}_{-1})_{\mathrm{In}}, (\tau^1{}_{-1})_{\mathrm{Out}}\right) + 0.0255966I\left((\tau^2{}_{-1})_{\mathrm{In}}, (\tau^1{}_1)_{\mathrm{Out}}\right)\\
&+ 0.0255966I\left((\tau^2{}_1)_{\mathrm{In}}, (\tau^1{}_{-1})_{\mathrm{Out}}\right) + 0.0255966I\left((\tau^2{}_1)_{\mathrm{In}}, (\tau^1{}_1)_{\mathrm{Out}}\right)\\
&- 0.00767384I\left((\tau^2{}_{-1})_{\mathrm{In}}, (\tau^1{}_{-1} + \mathrm{i}\tau^1{}_0)_{\mathrm{Out}}\right) + 0.00767384I\left((\tau^2{}_1)_{\mathrm{In}}, (\tau^1{}_{-1} + \mathrm{i}\tau^1{}_0)_{\mathrm{Out}}\right)\\
&+ 0.00770635I\left((\tau^2{}_{-1})_{\mathrm{In}}, (\tau^1{}_{+1} + \mathrm{i}\tau^1{}_{-1})_{\mathrm{Out}}\right) + 0.00770635I\left((\tau^2{}_1)_{\mathrm{In}}, (\tau^1{}_{+1} + \mathrm{i}\tau^1{}_{-1})_{\mathrm{Out}}\right)\\
&+ 0.00767384I\left((\tau^2{}_{-1})_{\mathrm{In}}, (\tau^1{}_{-1} + \tau^1{}_0)_{\mathrm{Out}}\right) - 0.00767384I\left((\tau^2{}_1)_{\mathrm{In}}, (\tau^1{}_{-1} + \tau^1{}_0)_{\mathrm{Out}}\right)\\
&+ 0.00770635I\left((\tau^2{}_{-1})_{\mathrm{In}}, (\tau^1{}_{+1} + \tau^1{}_{-1})_{\mathrm{Out}}\right) + 0.00770635I\left((\tau^2{}_1)_{\mathrm{In}}, (\tau^1{}_{+1} + \tau^1{}_{-1})_{\mathrm{Out}}\right)\\
&- 0.00444926I\left((\tau^2{}_{-1} + \mathrm{i}\tau^2{}_0)_{\mathrm{In}}, (\tau^1{}_0 + \mathrm{i}\tau^1{}_{+1})_{\mathrm{Out}}\right) - 0.00222463I\left((\tau^2{}_{-1} + \mathrm{i}\tau^2{}_0)_{\mathrm{In}}, (\tau^1{}_{-1})_{\mathrm{Out}}\right)\\
&+ 0.00222463I\left((\tau^2{}_{-1} + \mathrm{i}\tau^2{}_0)_{\mathrm{In}}, (\tau^1{}_1)_{\mathrm{Out}}\right) + 0.00444926I\left((\tau^2{}_{-1} + \mathrm{i}\tau^2{}_0)_{\mathrm{In}}, (\tau^1{}_{-1} + \mathrm{i}\tau^1{}_0)_{\mathrm{Out}}\right)\\
&+ 0.00770635I\left((\tau^2{}_{-1} + \mathrm{i}\tau^2{}_1)_{\mathrm{In}}, (\tau^1{}_{-1})_{\mathrm{Out}}\right) + 0.00770635I\left((\tau^2{}_{-1} + \mathrm{i}\tau^2{}_1)_{\mathrm{In}}, (\tau^1{}_1)_{\mathrm{Out}}\right)\\
&- 0.0154127I\left((\tau^2{}_{-1} + \mathrm{i}\tau^2{}_1)_{\mathrm{In}}, (\tau^1{}_{+1} + \mathrm{i}\tau^1{}_{-1})_{\mathrm{Out}}\right) + 0.0108984I\left((\tau^2{}_1 + \mathrm{i}\tau^2{}_2)_{\mathrm{In}}, (\tau^1{}_0 + \mathrm{i}\tau^1{}_{+1})_{\mathrm{Out}}\right)\\
&+ 0.00544921I\left((\tau^2{}_1 + \mathrm{i}\tau^2{}_2)_{\mathrm{In}}, (\tau^1{}_{-1})_{\mathrm{Out}}\right) - 0.00544921I\left((\tau^2{}_1 + \mathrm{i}\tau^2{}_2)_{\mathrm{In}}, (\tau^1{}_1)_{\mathrm{Out}}\right)\\
&- 0.0108984I\left((\tau^2{}_1 + \mathrm{i}\tau^2{}_2)_{\mathrm{In}}, (\tau^1{}_{-1} + \mathrm{i}\tau^1{}_0)_{\mathrm{Out}}\right) + 0.00444926I\left((\tau^2{}_{-1} + \tau^2{}_0)_{\mathrm{In}}, (\tau^1{}_0 + \tau^1{}_{+1})_{\mathrm{Out}}\right)\\
&+ 0.00222463I\left((\tau^2{}_{-1} + \tau^2{}_0)_{\mathrm{In}}, (\tau^1{}_{-1})_{\mathrm{Out}}\right) - 0.00222463I\left((\tau^2{}_{-1} + \tau^2{}_0)_{\mathrm{In}}, (\tau^1{}_1)_{\mathrm{Out}}\right)\\
&- 0.00444926I\left((\tau^2{}_{-1} + \tau^2{}_0)_{\mathrm{In}}, (\tau^1{}_{-1} + \tau^1{}_0)_{\mathrm{Out}}\right) + 0.00770635I\left((\tau^2{}_{-1} + \tau^2{}_1)_{\mathrm{In}}, (\tau^1{}_{-1})_{\mathrm{Out}}\right)\\
&+ 0.00770635I\left((\tau^2{}_{-1} + \tau^2{}_1)_{\mathrm{In}}, (\tau^1{}_1)_{\mathrm{Out}}\right) - 0.0154127I\left((\tau^2{}_{-1} + \tau^2{}_1)_{\mathrm{In}}, (\tau^1{}_{+1} + \tau^1{}_{-1})_{\mathrm{Out}}\right)\\
&- 0.0108984I\left((\tau^2{}_1 + \tau^2{}_2)_{\mathrm{In}}, (\tau^1{}_0 + \tau^1{}_{+1})_{\mathrm{Out}}\right) - 0.00544921I\left((\tau^2{}_1 + \tau^2{}_2)_{\mathrm{In}}, (\tau^1{}_{-1})_{\mathrm{Out}}\right)\\
&+ 0.00544921I\left((\tau^2{}_1 + \tau^2{}_2)_{\mathrm{In}}, (\tau^1{}_1)_{\mathrm{Out}}\right) + 0.0108984I\left((\tau^2{}_1 + \tau^2{}_2)_{\mathrm{In}}, (\tau^1{}_{-1} + \tau^1{}_0)_{\mathrm{Out}}\right)\\
&+ 0.0371561I\left((\tau^2{}_{-2})_{\mathrm{In}}, (\tau^1{}_0)_{\mathrm{Out}}\right) + 0.0371561I\left((\tau^2{}_2)_{\mathrm{In}}, (\tau^1{}_0)_{\mathrm{Out}}\right)\\
&+ 0.00544921I\left((\tau^2{}_{-2})_{\mathrm{In}}, (\tau^1{}_0 + \mathrm{i}\tau^1{}_{+1})_{\mathrm{Out}}\right) - 0.00544921I\left((\tau^2{}_2)_{\mathrm{In}}, (\tau^1{}_0 + \mathrm{i}\tau^1{}_{+1})_{\mathrm{Out}}\right)\\
&- 0.00544921I\left((\tau^2{}_{-2})_{\mathrm{In}}, (\tau^1{}_0 + \tau^1{}_{+1})_{\mathrm{Out}}\right) + 0.00544921I\left((\tau^2{}_2)_{\mathrm{In}}, (\tau^1{}_0 + \tau^1{}_{+1})_{\mathrm{Out}}\right)\\
&+ 0.0231575I\left((\tau^2{}_{-2})_{\mathrm{In}}, (\tau^1{}_{-1})_{\mathrm{Out}}\right) + 0.0231575I\left((\tau^2{}_{-2})_{\mathrm{In}}, (\tau^1{}_1)_{\mathrm{Out}}\right)\\
&+ 0.0231575I\left((\tau^2{}_2)_{\mathrm{In}}, (\tau^1{}_{-1})_{\mathrm{Out}}\right) + 0.0231575I\left((\tau^2{}_2)_{\mathrm{In}}, (\tau^1{}_1)_{\mathrm{Out}}\right)\\
&- 0.00544921I\left((\tau^2{}_{-2})_{\mathrm{In}}, (\tau^1{}_{-1} + \mathrm{i}\tau^1{}_0)_{\mathrm{Out}}\right) + 0.00544921I\left((\tau^2{}_2)_{\mathrm{In}}, (\tau^1{}_{-1} + \mathrm{i}\tau^1{}_0)_{\mathrm{Out}}\right)\\
&+ 0.00629221I\left((\tau^2{}_{-2})_{\mathrm{In}}, (\tau^1{}_{+1} + \mathrm{i}\tau^1{}_{-1})_{\mathrm{Out}}\right) + 0.00629221I\left((\tau^2{}_2)_{\mathrm{In}}, (\tau^1{}_{+1} + \mathrm{i}\tau^1{}_{-1})_{\mathrm{Out}}\right)\\
&+ 0.00544921I\left((\tau^2{}_{-2})_{\mathrm{In}}, (\tau^1{}_{-1} + \tau^1{}_0)_{\mathrm{Out}}\right) - 0.00544921I\left((\tau^2{}_2)_{\mathrm{In}}, (\tau^1{}_{-1} + \tau^1{}_0)_{\mathrm{Out}}\right)\\
&+ 0.00629221I\left((\tau^2{}_{-2})_{\mathrm{In}}, (\tau^1{}_{+1} + \tau^1{}_{-1})_{\mathrm{Out}}\right) + 0.00629221I\left((\tau^2{}_2)_{\mathrm{In}}, (\tau^1{}_{+1} + \tau^1{}_{-1})_{\mathrm{Out}}\right)\\
&+ 0.00629221I\left((\tau^2{}_{-2} + \mathrm{i}\tau^2{}_0)_{\mathrm{In}}, (\tau^1{}_{-1})_{\mathrm{Out}}\right) + 0.00629221I\left((\tau^2{}_{-2} + \mathrm{i}\tau^2{}_0)_{\mathrm{In}}, (\tau^1{}_1)_{\mathrm{Out}}\right)\\
&- 0.0125844I\left((\tau^2{}_{-2} + \mathrm{i}\tau^2{}_0)_{\mathrm{In}}, (\tau^1{}_{+1} + \mathrm{i}\tau^1{}_{-1})_{\mathrm{Out}}\right) - 0.0108984I\left((\tau^2{}_{-2} + \mathrm{i}\tau^2{}_{-1})_{\mathrm{In}}, (\tau^1{}_0 + \mathrm{i}\tau^1{}_{+1})_{\mathrm{Out}}\right)\\
&- 0.00544921I\left((\tau^2{}_{-2} + \mathrm{i}\tau^2{}_{-1})_{\mathrm{In}}, (\tau^1{}_{-1})_{\mathrm{Out}}\right) + 0.00544921I\left((\tau^2{}_{-2} + \mathrm{i}\tau^2{}_{-1})_{\mathrm{In}}, (\tau^1{}_1)_{\mathrm{Out}}\right)\\
&+ 0.0108984I\left((\tau^2{}_{-2} + \mathrm{i}\tau^2{}_{-1})_{\mathrm{In}}, (\tau^1{}_{-1} + \mathrm{i}\tau^1{}_0)_{\mathrm{Out}}\right) + 0.00629221I\left((\tau^2{}_{-2} + \tau^2{}_0)_{\mathrm{In}}, (\tau^1{}_{-1})_{\mathrm{Out}}\right)\\
&+ 0.00629221I\left((\tau^2{}_{-2} + \tau^2{}_0)_{\mathrm{In}}, (\tau^1{}_1)_{\mathrm{Out}}\right) - 0.0125844I\left((\tau^2{}_{-2} + \tau^2{}_0)_{\mathrm{In}}, (\tau^1{}_{+1} + \tau^1{}_{-1})_{\mathrm{Out}}\right)\\
&+ 0.0108984I\left((\tau^2{}_{-2} + \tau^2{}_{-1})_{\mathrm{In}}, (\tau^1{}_0 + \tau^1{}_{+1})_{\mathrm{Out}}\right) + 0.00544921I\left((\tau^2{}_{-2} + \tau^2{}_{-1})_{\mathrm{In}}, (\tau^1{}_{-1})_{\mathrm{Out}}\right)\\
&- 0.00544921I\left((\tau^2{}_{-2} + \tau^2{}_{-1})_{\mathrm{In}}, (\tau^1{}_1)_{\mathrm{Out}}\right) - 0.0108984II\left((\tau^2{}_{-2} + \tau^2{}_{-1})_{\mathrm{In}}, (\tau^1{}_{-1} + \tau^1{}_0)_{\mathrm{Out}}\right)
\end{aligned}
$$

3 Additional Supplementary Data

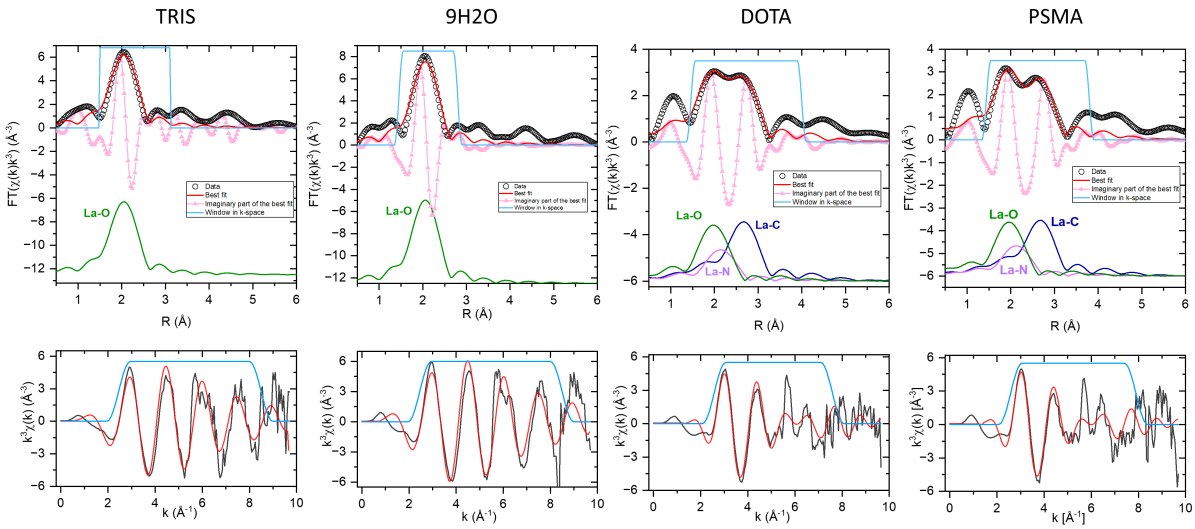

Supplementary Figure 1. left to right) R-space fit results for TRIS, 9H2O, DOTA and PSMA. In the upper panels, the magnitudes of the FT-EXAFS (black circles), their best fits (red lines), the imaginary parts of FT-EXAFS (pink triangles), and the single scattering paths (green, violet and blue lines) are shown. In the lower panels, k^3-weighted filtered $\chi(k)$ function (black lines) and their best fits (red lines) are also shown

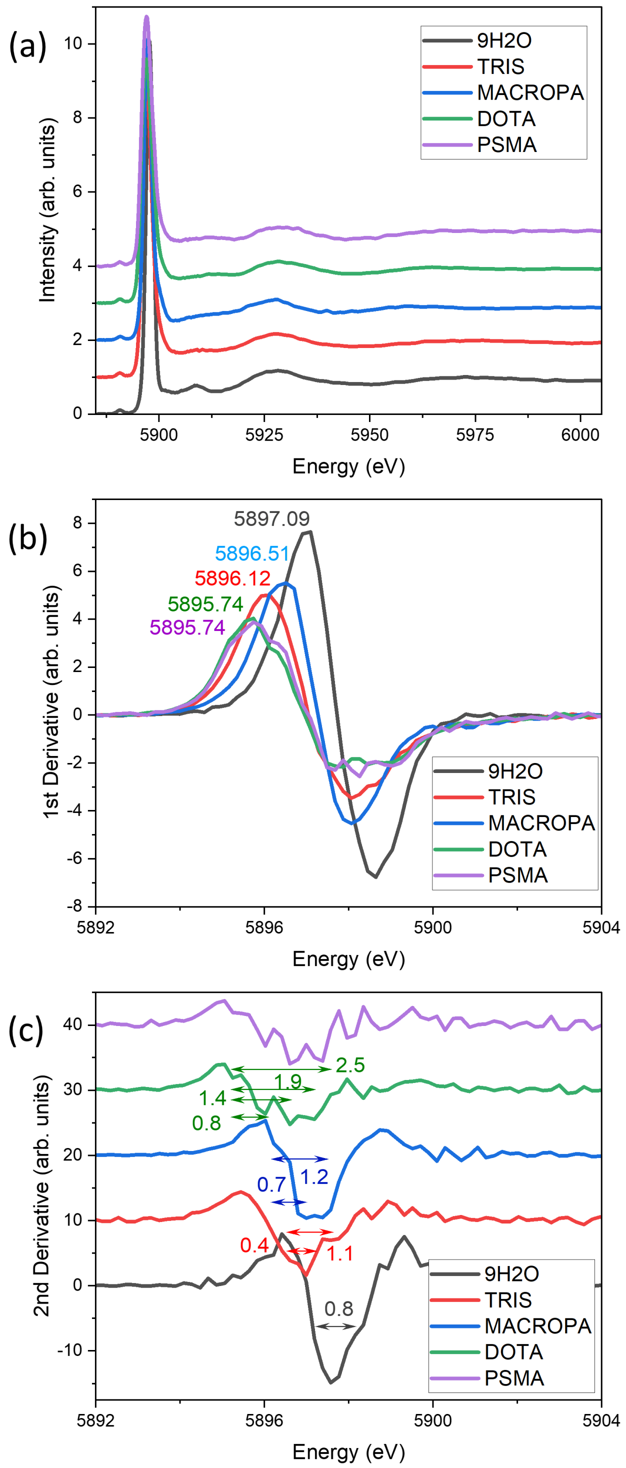

Supplementary Figure 2. a) Normalized La L_2-edge HR-XANES spectra of 9H2O, TRIS, MACROPA, DOTA and PSMA. b) First derivative of the HR-XANES spectra. The energy values of the maximum of the first derivative data are highlighted, they represent the inflection point of the main absorption edge. c) Second derivative of the HR-XANES spectra, showing some specific features related to the $5d$ ligand-field splitting. The experimentally derived $5d$ ligand-field strength are also shown.

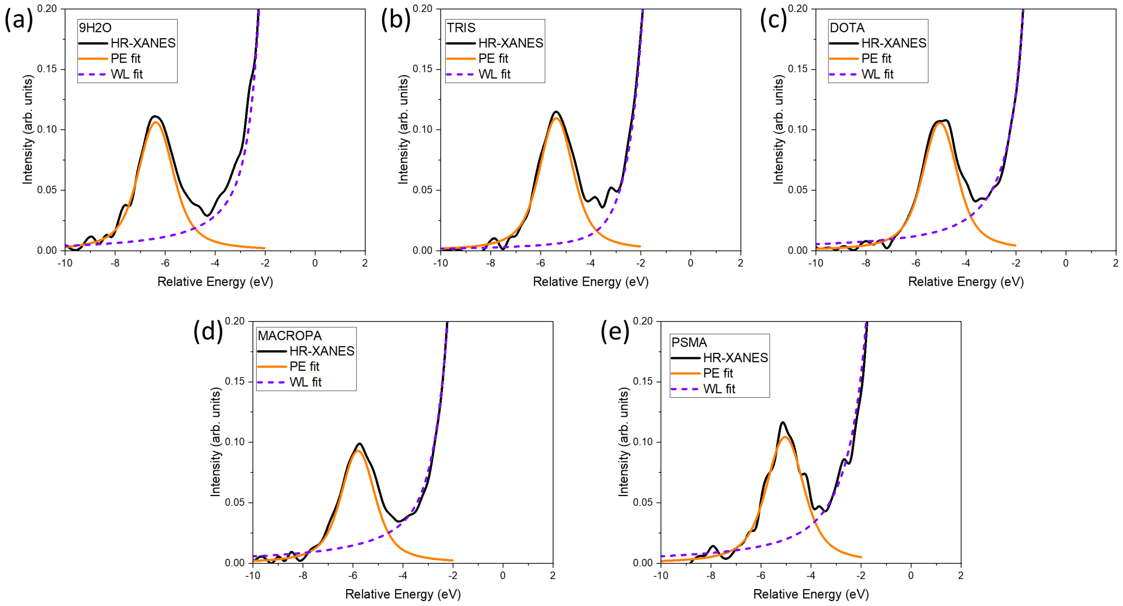

Supplementary Figure 3. a-b-c-d-e) Fit to pseudo-Voigt functions (orange lines) of the pre-edge energy region of the La L_2-edge HR-XANES spectra of 9H2O, TRIS, MACROPA, DOTA, and PSMA. The energy axis is represented relative to the inflection point of the main absorption edge (maximum of the first-derivative in Figure 2(b)).

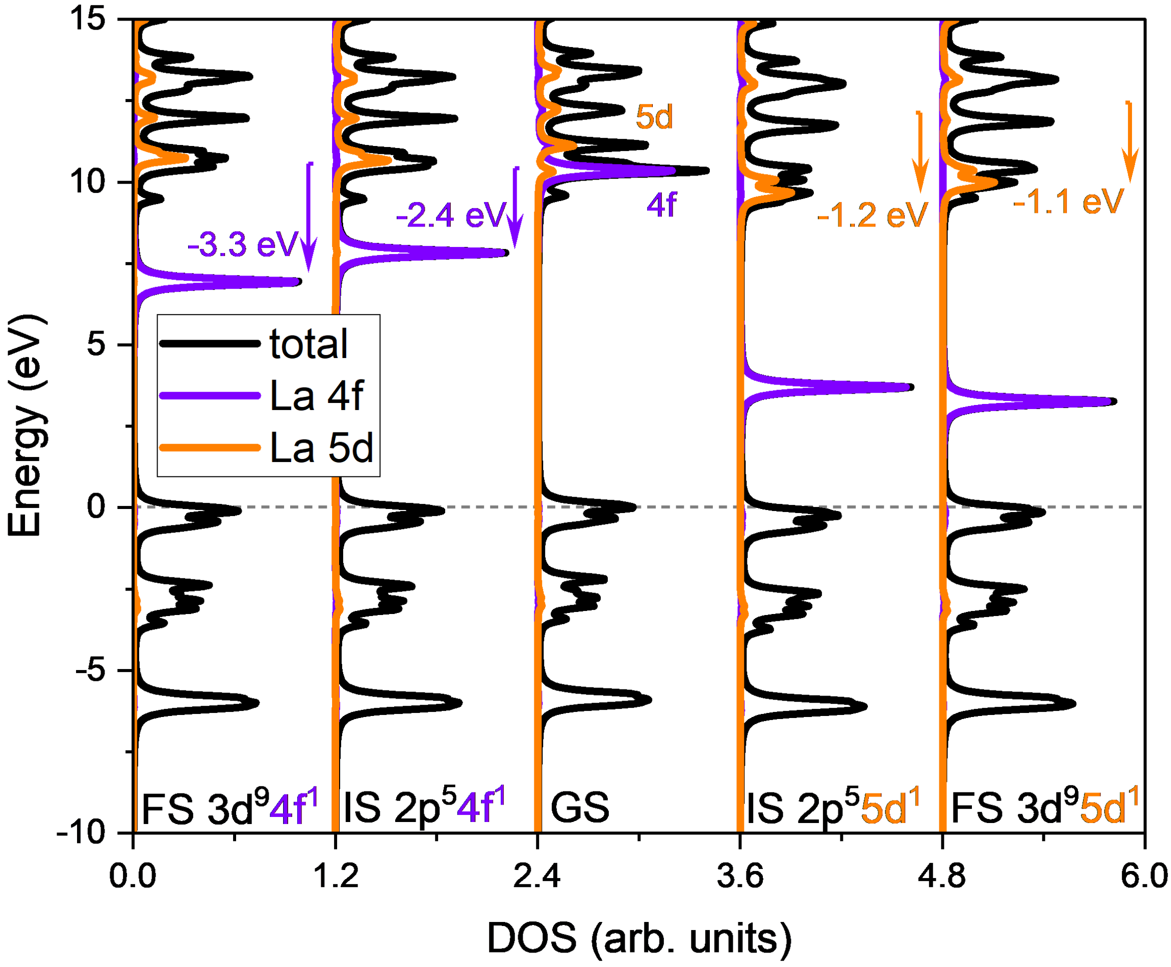

Supplementary Figure 4. Electronic structures of 9H2O as function of the GS, IS ($2p^54f^1$ and $2p^55d^1$) and FS ($3d^94f^1$ and $3d^95d^1$), showing the calculated total density of states (DOS) and angular-momentum projected DOS for La f and d orbitals. The electronic structure is selectively shown in the energy region that correspond to the frontier molecular orbitals. The calculations are done using DFT with the PBE0[6] functionals based on the optimized structures obtained with the same functional. The energy of the highest occupied molecular orbitals is set to 0 eV in GS, IS and FS. Note that in the IS and FS, core-holes are present in $2p$ and $3d$, respectively, where one additional electron is placed in the $4f$ and $5d$ orbitals. The resulting open-shell electrons are treated with the average of configuration type calculations (see the Main Text for details). The energy stabilization due to the presence of the core-hole is highlighted in the graph.

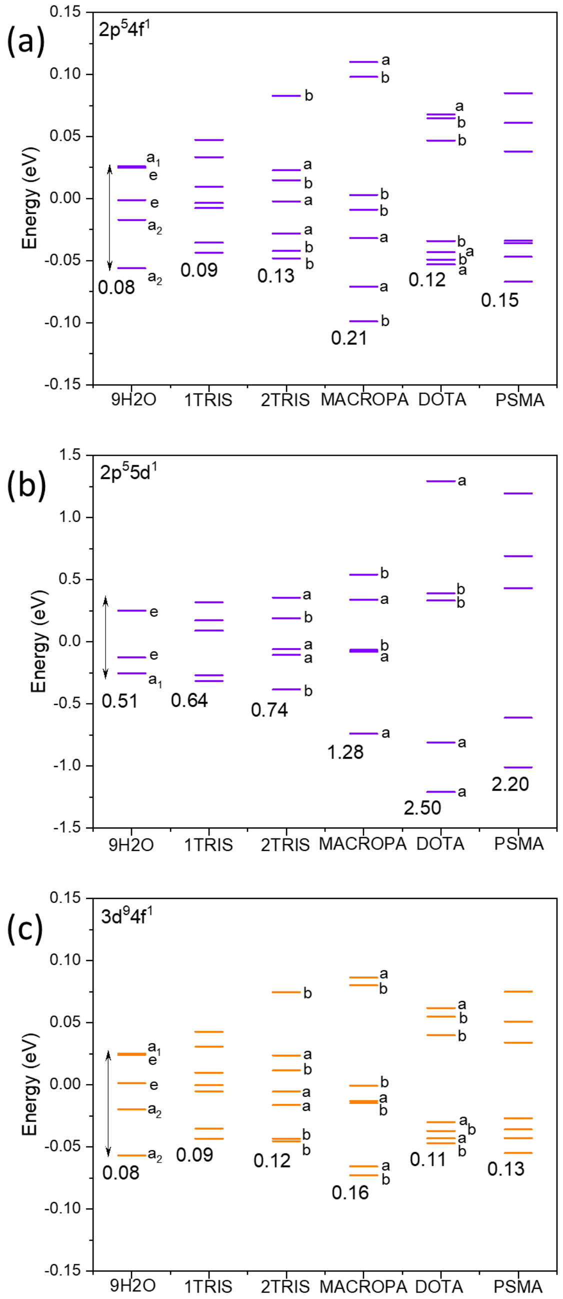

Supplementary Figure 5. a) Representation of the calculated La 4*f* ligand-field potential of 9H2O, 1TRIS, 2TRIS, MACROPA, DOTA and PSMA, showing the energy splitting in IS. b) Representation of the calculated La 5*d* ligand-field potential of 9H2O, 1TRIS, 2TRIS, MACROPA, DOTA and PSMA, showing the energy splitting in IS. c) same as in (a) with the energy splitting obtained in FS. The calculations are done using LFDFT with the PBE0[6] DFT functional based on the optimized structures obtained with the same functional. The extraction of the parameters from the DFT calculations can be seen elsewhere.[7] The relative strengths of the ligand-field potential are highlighted (in eV), revealing that MACROPA has the strongest 4*f* ligand-field splitting, whereas DOTA/PSMA has the strongest 5*d*. Symmetry representations are also shown for 9H2O (D_3 point group) and 2TRIS, MACROPA and DOTA (C_2 point group). Calculated ligand-field potential of the the La 4*f* electrons in the FS is also represented for comparison (c). This shows only negligible differences between the IS and FS electronic structures.

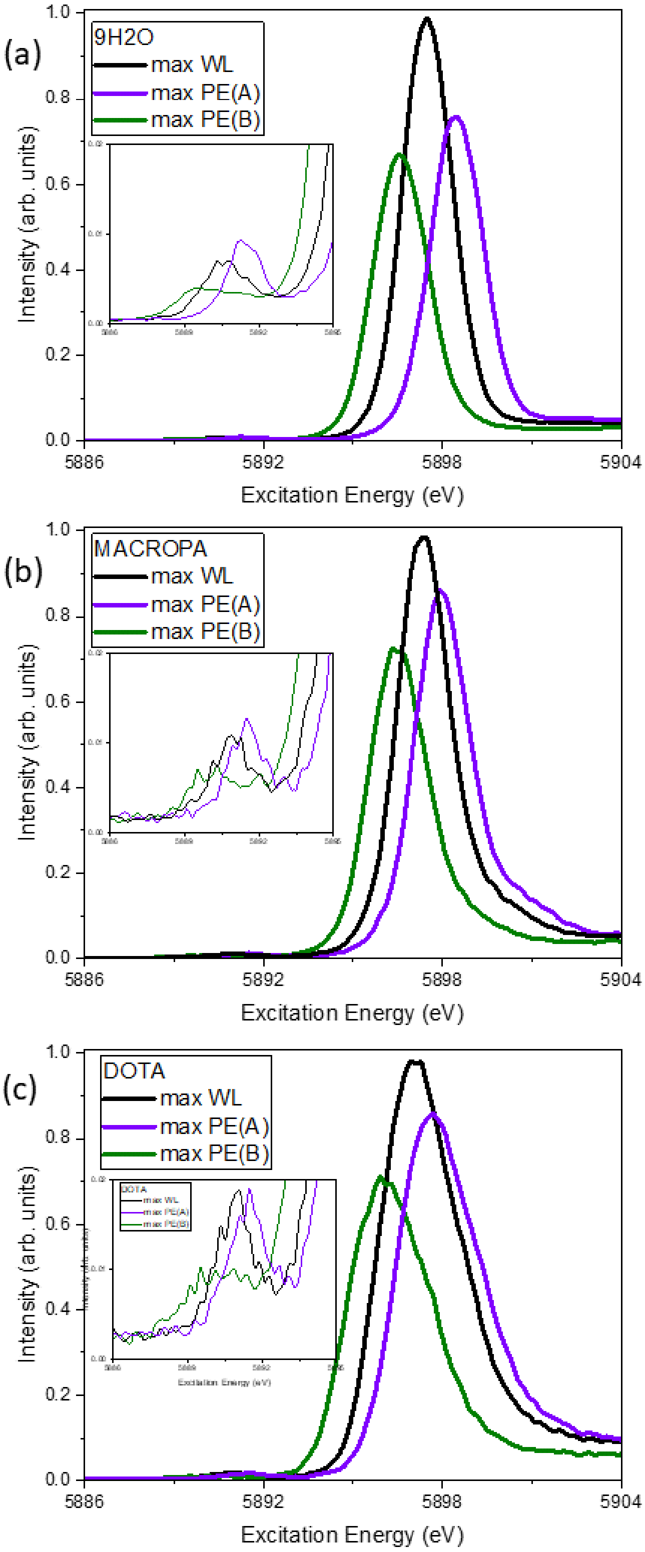

Supplementary Figure 6. Cross sections of the experimental CC-RIXS maps of 9H2O (a), MACROPA (b) and DOTA (c) along the excitation energy (see also in the main text Figure 4 d-f) performed at the emission energies that correspond to the maximum of the main absorption peak (WL), pre-edge (PE) peak A and peak B.

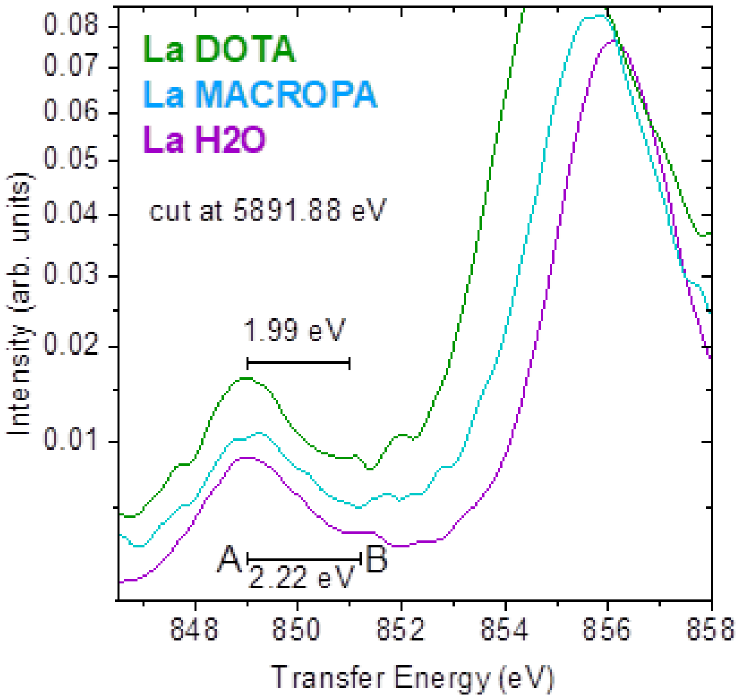

Supplementary Figure 7. Cross sections of the experimental CC-RIXS maps of 9H2O, MACROPA and DOTA along the transfer energy (see also in the main text Figure 4 d-f) performed at the excitation energies that correspond to the pre-edge (PE) peak, i.e. 5891.88 eV

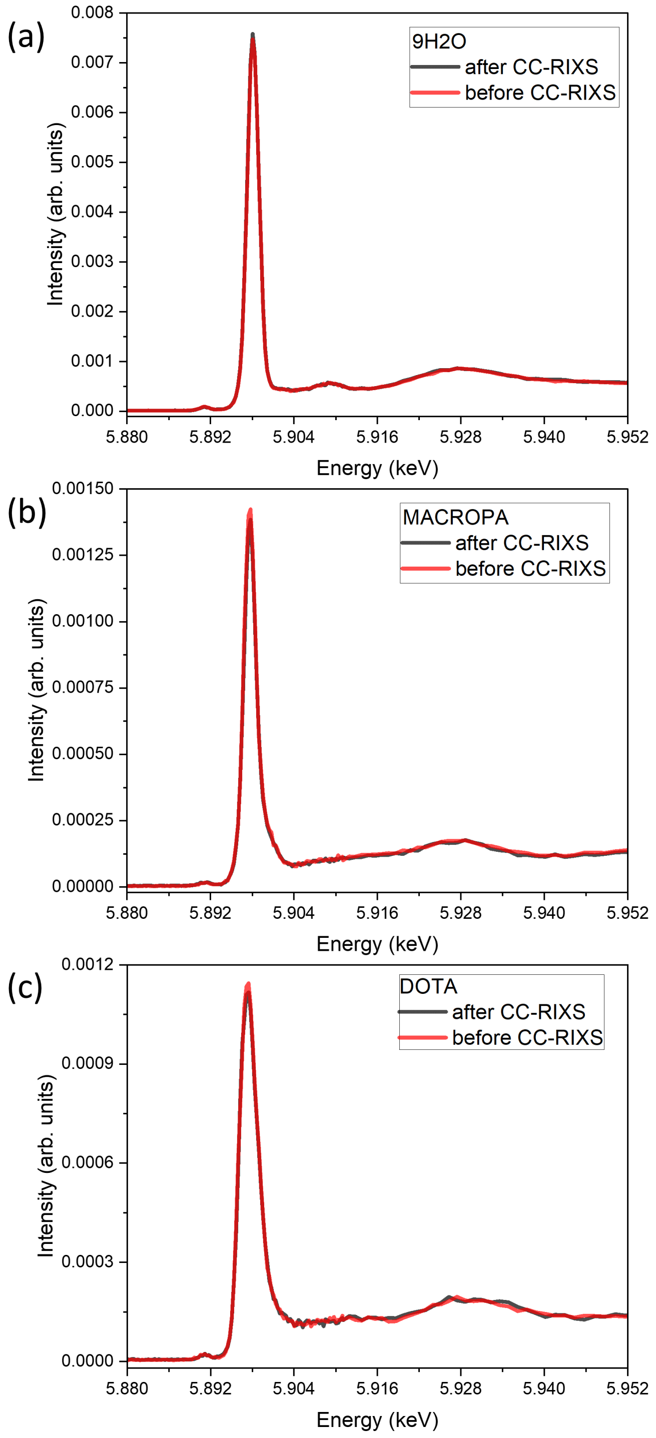

Supplementary Figure 8. La L_2-edge HR-XANES spectra of 9H2O (a), MACROPA (b) and DOTA (c) before and after the La L_2-edge CC-RIXS experiments showing no radiation damage during the measurements.

Supplementary Table 1. EXAFS fit results for 9H2O, TRIS, DOTA and PSMA: single-scattering path (SP), coordination number (N_i), path length (R_i in Å), mean-square displacement of the bond length or Debye-Waller factor (σ_i^2 in Å^2), energy shift of the ionization potential (ΔE_0 in eV), passive electron reduction factor (S_0^2), and goodness of fit (r in χ^2).

Complex	SP	N_i[a]	R_i	σ_i^2	ΔE_0	S_0^2	r
9H2O	La-O	11±2	2.57±0.03	0.011±0.005	2.3±1.5	1	0.015
TRIS	La-O	10±2	2.58±0.02	0.012±0.004	1.6±1.3	1	0.017
DOTA	La-O	5	2.53±0.03				
	La-N	4	2.74±0.04	0.011±0.003	7.6±1.3	1	0.017
	La-C	16	3.37±0.03				
PSMA	La-O	5	2.51±0.02				
	La-N	4	2.71±0.03	0.013±0.002	7.6±1.1	1	0.014
	La-C	16	3.37±0.03				

[a]N is optimized for 9H2O and TRIS, but frozen otherwise.

Supplementary Table 2. Cartesian coordinates for 9H2O optimized structures (in Å)

PBE			
La	0.00000000000000	0.00000000000000	0.00000000000000
O	-1.27671236164797	-2.21133068800572	0.00000000000000
O	-1.27671236164797	2.21133068800572	0.00000000000000
O	2.55342473329593	-0.00000000000000	0.00000000000000
O	0.88682555170946	-1.58004134329279	-1.79893580512050
O	0.92494316524273	-1.55803412507737	1.79893580512050
O	0.92494316524273	1.55803412507737	-1.79893580512050
O	0.88682555170946	1.58004134329279	1.79893580512050
O	-1.81176871695219	0.02200721821542	-1.79893580512050
O	-1.81176871695219	-0.02200721821542	1.79893580512050
H	-1.06203737516836	-3.01014905026279	-0.51933981376995
H	-2.07584685806425	-2.42482587842137	0.51933981376995
H	-2.40466456430472	0.74464782416248	1.92311762787211
H	-1.79054096929568	-0.48437847027774	2.65912610856115
H	-2.40466456430472	-0.74464782416248	-1.92311762787211
H	-1.79054096929568	0.48437847027774	-2.65912610856115
H	1.84721621056458	1.71017669066705	1.92311762787211
H	0.47578642283761	1.79284320577517	2.65912610856115
H	0.55744835374014	2.45482450482952	-1.92311762787211
H	1.31475454645808	1.30846472549743	-2.65912610856115
H	0.55744835374014	-2.45482450482952	1.92311762787211
H	1.31475454645808	-1.30846472549743	2.65912610856115
H	1.84721621056458	-1.71017669066705	-1.92311762787211
H	0.47578642283761	-1.79284320577517	-2.65912610856115
H	3.13788424323260	-0.58532318184142	0.51933981376995
H	3.13788424323260	0.58532318184142	-0.51933981376995
H	-1.06203737516836	3.01014905026279	0.51933981376995
H	-2.07584685806425	2.42482587842137	-0.51933981376995
PBE0			
La	0.00000000000000	0.00000000000000	0.00000000000000
O	-1.26804521763148	-2.19631875421264	0.00000000000000
O	-1.26804521763148	2.19631875421264	0.00000000000000
O	2.53609044526296	-0.00000000000000	0.00000000000000
O	0.87901519730824	-1.56893423058173	-1.79119698776684
O	0.91922930067287	-1.54571660339783	1.79119698776684
O	0.91922930067287	1.54571660339783	-1.79119698776684
O	0.87901519730824	1.56893423058173	1.79119698776684
O	-1.79824449798110	0.02321762718391	-1.79119698776684
O	-1.79824449798110	-0.02321762718391	1.79119698776684
H	-1.05502107957511	-2.98608478156251	-0.51428347395399
H	-2.05851473784293	-2.40671745384701	0.51428347395399
H	-2.40481070138195	0.72031246260963	1.90649050133780
H	-1.79132302984565	-0.49477026980803	2.63454142316321
H	-2.40481070138195	-0.72031246260963	-1.90649050133780
H	-1.79132302984565	0.49477026980803	-2.63454142316321
H	1.82621423778815	1.72247092986479	1.90649050133780
H	0.46717789072833	1.79871638984388	2.63454142316321
H	0.57859646359380	2.44278338247442	-1.90649050133780
H	1.32414513911732	1.30394611003585	-2.63454142316321
H	0.57859646359380	-2.44278338247442	1.90649050133780
H	1.32414513911732	-1.30394611003585	2.63454142316321
H	1.82621423778815	-1.72247092986479	-1.90649050133780
H	0.46717789072833	-1.79871638984388	-2.63454142316321
H	3.11353582741803	-0.57936733771549	0.51428347395399
H	3.11353582741803	0.57936733771549	-0.51428347395399

H	-1.05502107957511	2.98608478156251	0.51428347395399
H	-2.05851473784293	2.40671745384701	-0.51428347395399
TPSSH			
La	0.00000000000000	0.00000000000000	0.00000000000000
O	-1.27445514018599	-2.20742106574964	0.00000000000000
O	-1.27445514018599	2.20742106574964	0.00000000000000
O	2.54891029037198	-0.00000000000000	0.00000000000000
O	0.86681032170506	-1.56026211528923	-1.82796651999154
O	0.91782146632660	-1.53081081342919	1.82796651999154
O	0.91782146632660	1.53081081342919	-1.82796651999154
O	0.86681032170506	1.56026211528923	1.82796651999154
O	-1.78463178803165	0.02945130186004	-1.82796651999154
O	-1.78463178803165	-0.02945130186004	1.82796651999154
H	-1.05752755060072	-3.00308441954741	-0.51127939576676
H	-2.07198362068019	-2.41738794042150	0.51127939576676
H	-2.40865233502350	0.70397297802307	1.94886155801576
H	-1.76443914369361	-0.50521205751893	2.67334190605724
H	-2.40865233502350	-0.70397297802307	-1.94886155801576
H	-1.76443914369361	0.50521205751893	-2.67334190605724
H	1.81398464587222	1.73396762448368	1.94886155801576
H	0.44469309423374	1.78065515533921	2.67334190605724
H	0.59466768915128	2.43794059250676	-1.94886155801576
H	1.31974604945987	1.27544308782029	-2.67334190605724
H	0.59466768915128	-2.43794059250676	1.94886155801576
H	1.31974604945987	-1.27544308782029	2.67334190605724
H	1.81398464587222	-1.73396762448368	-1.94886155801576
H	0.44469309423374	-1.78065515533921	-2.67334190605724
H	3.12951118128091	-0.58569648912591	0.51127939576676
H	3.12951118128091	0.58569648912591	-0.51127939576676
H	-1.05752755060072	3.00308441954741	0.51127939576676
H	-2.07198362068019	2.41738794042150	-0.51127939576676

Supplementary Table 3. Cartesian coordinates for 1TRIS optimized structures (in Å)

PBE			
La	0.00807711202005	-0.00297514304569	0.11287460073912
O	1.94427981285785	1.18427661413006	1.25256521645290
O	1.78731896937440	-1.63071931581098	0.89429784565623
O	-0.78660596677446	1.23347630140172	2.19097120935764
O	-0.11067742880376	2.50930824777372	-0.33599901147704
O	-0.45828766695737	0.27485528701532	-2.41106933798102
O	-0.94160636468138	-1.72550350067826	1.76471151521696
O	-0.73025886095931	-2.12242030028353	-1.08272261096707
O	-2.50399057256244	0.24635874230427	-0.27668497387446
N	2.34566920007272	0.13540376552535	-1.16258745214772
H	1.87969410706171	1.45753396235746	2.18818437275976
H	-1.89221569664680	-1.94706392205295	1.81157291461228
H	-1.07197242583765	2.16805486483168	2.19529198688643
H	1.58149044552083	-2.56805089469733	1.07926880479376
H	2.52444412248311	1.05613709690799	-1.58314131958225
H	-3.08491314533202	-0.43791188373942	-0.66293430130606
H	2.42822840992883	-0.53844724982611	-1.93426255763053
H	-0.55778890706867	2.96640290132095	-1.07405947998279
H	-0.79160462313472	-3.03799621721378	-0.74956531618843
H	-1.34320994685286	0.58828724219754	-2.68627991712941
H	-1.25599689412898	0.80646888414055	2.93359903022739
H	0.58893358712618	3.11959090422335	-0.03140784507621

H	-0.72723733730429	-2.18245927921609	-2.05788009654378
H	0.15745116774805	0.66797955803448	-3.05987137709442
H	-0.58029185709129	-1.96579413771863	2.64021616150637
H	-3.09528142011186	0.82543187319170	0.24279258114194
C	3.40674697958982	-0.15398218426904	-0.15692562556807
C	3.15271999065019	-1.55820565460403	0.39034599717727
C	3.33903544046372	0.94095568792043	0.91571238920000
H	3.27366282254636	-2.29462677096424	-0.41712008031827
H	3.76686877750834	1.87495190286290	0.52264625399626
H	3.89242127328669	0.63831264809842	1.81046931211074
H	3.85400715967174	-1.78603259572762	1.20155559969860
C	4.79516193290751	-0.12507323003150	-0.81587593601951
H	4.93891276249062	0.86182912607791	-1.28703528517951
H	4.83482049479217	-0.89763396326336	-1.60260688919000
O	5.78217405481355	-0.36436027554965	0.20009478986752
H	6.65476349133338	-0.32609909162361	-0.23598616814458
PBE0			
La	0.01724210920453	-0.00660872391966	0.09892324201164
O	1.94064859443311	1.16221240119138	1.24347093263700
O	1.79187898240291	-1.63067798642332	0.85842887278793
O	-0.78307976135922	1.21720463316393	2.16434752285769
O	-0.10048610432262	2.48903149411390	-0.34630405211053
O	-0.45729541438356	0.27371139438790	-2.40272563908682
O	-0.89855013306208	-1.71560403541313	1.75753401935519
O	-0.74242981926714	-2.11319924253871	-1.07169465655341
O	-2.48369494130397	0.26317837868680	-0.26810276874985
N	2.34824321095243	0.13505953747760	-1.15778474173022
H	1.86958509662715	1.46167826880002	2.15887238635852
H	-1.83336154928619	-1.95563839111666	1.80946263709908
H	-1.04605227420668	2.14668057196405	2.18158814964964
H	1.59366951108383	-2.55125029837950	1.07396002347364
H	2.52136381176969	1.04926781579644	-1.57202251406143
H	-3.06904497050574	-0.39828605465871	-0.66003302251896
H	2.43089396951264	-0.53107240794675	-1.92357179882072
H	-0.56745894203894	2.94711762110384	-1.05665130567360
H	-0.81831249928654	-3.01239009698761	-0.72872697926234
H	-1.33268593189778	0.57466211252890	-2.68310738581048
H	-1.22944493238799	0.79796080081842	2.91136965021341
H	0.57800903351199	3.10270018419477	-0.03552408273529
H	-0.76109516873919	-2.18173653301953	-2.03505201767168
H	0.15387294377189	0.63506312625216	-3.05803112562939
H	-0.52459338897807	-1.95527539191136	2.61570558183934
H	-3.05862859724567	0.85423889695294	0.23562675453259
C	3.39400648558422	-0.15228263382829	-0.15766531701127
C	3.14349318601245	-1.55061410675243	0.37919955775569
C	3.31796982049844	0.92783897974594	0.91499352667860
H	3.28049435644688	-2.27740265882575	-0.42404615902458
H	3.74646658502675	1.85678844805076	0.53287886740206
H	3.86639310282532	0.62189807895549	1.80245872934146
H	3.83444719498984	-1.77543225365471	1.18946400391266
C	4.77751201885103	-0.11754110212966	-0.79776800306557
H	4.92616617196688	0.86501834174740	-1.25711530351533
H	4.82538079190060	-0.87697472364610	-1.58527866430154
O	5.74082943792983	-0.36281101547056	0.21352300608763
H	6.61259101296897	-0.32625342931018	-0.19744692666073
TPSSH			
La	-0.00825860024965	0.01235256479231	0.12468794702165
O	1.93821856608261	1.09469390703831	1.32930723513604

O	1.76554783563971	-1.65841523197966	0.82672115010906
O	-0.88359831043138	1.33409106260847	2.11199136309277
O	0.05927492025859	2.52926061078074	-0.34530810562170
O	-0.48667793641457	0.36929580385870	-2.37409192024125
O	-0.91692049024422	-1.66110699922202	1.84091365036639
O	-0.69286712173036	-2.12995160017794	-1.08380355486383
O	-2.53699483405515	0.08561987453149	-0.27381882535469
N	2.31738897609486	0.16947026944721	-1.13563360572473
H	1.88508810426044	1.38501905439364	2.25331832939876
H	-1.85891929451647	-1.87351938997666	1.93673814928150
H	-1.13095927308839	2.27038550405127	2.04899453234391
H	1.55570459545205	-2.59087125890268	0.99375446568355
H	2.49096256119076	1.09766055034914	-1.52218231243676
H	-3.04104822334699	-0.66378033863169	-0.62921207454103
H	2.39052855325748	-0.47274992396879	-1.92538844201801
H	-0.24095191331751	2.98034100140665	-1.14964518785364
H	-0.70855657881085	-3.03190679505445	-0.72765989890562
H	-1.39185128034494	0.59164973961182	-2.64710499072202
H	-1.40206703111695	0.96648654366760	2.84466265941739
H	0.77333986413682	3.07722196724567	0.01714046112710
H	-0.62818473311026	-2.22001922941778	-2.04764023769640
H	0.09014226370088	0.77331811701517	-3.04118952374011
H	-0.51285775985910	-1.88147172235914	2.69530435646443
H	-3.17511117309924	0.62215116041502	0.22235207767802
C	3.38502515792076	-0.15614399569954	-0.15284817103071
C	3.13274215854409	-1.57525595400244	0.34263446186381
C	3.32670731752048	0.89006347170730	0.96083376960415
H	3.25651876067662	-2.28154201980450	-0.47856421439084
H	3.72817146627082	1.83918899459961	0.60396953075736
H	3.89033957569588	0.55838794406857	1.82785415870050
H	3.81538620703725	-1.82647969432928	1.15074512949040
C	4.75723434124697	-0.09726574787131	-0.83294803722745
H	4.89950863258250	0.90354540133679	-1.24965922361359
H	4.78342689680918	-0.82588527353042	-1.64827772613852
O	5.75860851034284	-0.38913845856085	0.14702282299079
H	6.62090228901444	-0.32443990943630	-0.29081519840666

Supplementary Table 4. Cartesian coordinates for 2TRIS optimized structures (in Å)

PBE			
La	0.00000000000000	-0.00000000000000	-0.01877992745384
N	-2.39819005049508	1.03071242906963	0.61479728251156
N	2.39819005049508	-1.03071242906963	0.61479728251156
O	-0.00000000000000	-0.00000000000000	2.50093008508424
O	-0.22373347226325	2.30677165358076	-1.10207001417365
O	0.22373347226325	-2.30677165358076	-1.10207001417365
O	-1.83818824940327	-1.60115884454907	0.77574056992118
O	1.83818824940327	1.60115884454907	0.77574056992118
O	-1.89728533204726	-0.28337495717861	-1.73210458088557
O	1.89728533204726	0.28337495717861	-1.73210458088557
O	5.81345541682985	0.31705081821201	-0.05413742156301
O	-5.81345541682985	-0.31705081821201	-0.05413742156301
C	-3.28683186337030	0.00911550414617	-1.41129381214040
C	3.28683186337030	-0.00911550414617	-1.41129381214040
C	-3.44007930807912	0.09959268437911	0.11031299635797
C	3.44007930807912	-0.09959268437911	0.11031299635797
C	-4.84748090932265	0.63123943958884	0.42950250915800

C	4.84748090932265	-0.63123943958884	0.42950250915800
C	-3.24743871058102	-1.24067063465288	0.82709665604896
C	3.24743871058102	1.24067063465288	0.82709665604896
H	-3.93198425780238	-0.78012329995486	-1.81486592608420
H	-3.56415742682114	0.97192080901215	-1.86602255462239
H	3.56415742682114	-0.97192080901215	-1.86602255462239
H	-3.54957366348361	-1.14104889681744	1.88030416845096
H	3.54957366348361	1.14104889681744	1.88030416845096
H	-3.84811280904450	-2.02257919479883	0.34848986908484
H	3.84811280904450	2.02257919479883	0.34848986908484
H	-4.97455960067716	1.61358974258463	-0.05624477862922
H	4.97455960067716	-1.61358974258463	-0.05624477862922
H	-4.93442309354866	0.76751997862445	1.52054813037843
H	4.93442309354866	-0.76751997862445	1.52054813037843
H	-6.69597165815790	0.04117783930198	0.16056511448149
H	6.69597165815790	-0.04117783930198	0.16056511448149
H	-2.49345129881812	1.13788023589355	1.63189779484199
H	2.49345129881812	-1.13788023589355	1.63189779484199
H	-2.55791022038177	1.96821865832618	0.22752105437918
H	2.55791022038177	-1.96821865832618	0.22752105437918
H	-1.80614690328798	-0.31212985373830	-2.70431055021668
H	1.80614690328798	0.31212985373830	-2.70431055021668
H	-1.73395662600824	-2.50337909555076	1.13455928344792
H	1.73395662600824	2.50337909555076	1.13455928344792
H	3.93198425780238	0.78012329995486	-1.81486592608420
H	0.49525906515957	0.60338549720578	3.08735251635354
H	-0.49525906515957	-0.60338549720578	3.08735251635354
H	0.86459673342898	-2.48936835755906	-1.81742786643781
H	0.62499669986095	2.66568770166492	-1.43128451947829
H	-0.86459673342898	2.48936835755906	-1.81742786643781
H	-0.62499669986095	-2.66568770166492	-1.43128451947829
PBE0			
La	-0.00000000000000	0.00000000000000	0.04675113812671
N	-2.39863333576052	1.01428134765797	0.63623628503843
N	2.39863333576052	-1.01428134765797	0.63623628503843
O	-0.00000000000000	0.00000000000000	2.55376125401839
O	-0.22303282582232	2.31284643342493	-1.00748790198467
O	0.22303282582232	-2.31284643342493	-1.00748790198467
O	-1.84877008375181	-1.58858108270904	0.79358022946486
O	1.84877008375181	1.58858108270904	0.79358022946486
O	-1.84009675095510	-0.26059236958605	-1.69982600008259
O	1.84009675095510	0.26059236958605	-1.69982600008259
O	5.75631755765198	0.31151262145374	-0.14851194930426
O	-5.75631755765198	-0.31151262145374	-0.14851194930426
C	-3.21733437971697	0.03118826845646	-1.41550002787707
C	3.21733437971697	-0.03118826845646	-1.41550002787707
C	-3.41375976090445	0.10005471089789	0.09186888746117
C	3.41375976090445	-0.10005471089789	0.09186888746117
C	-4.82089602672587	0.61941947820297	0.37148833302074
C	4.82089602672587	-0.61941947820297	0.37148833302074
C	-3.24227982653280	-1.24232954032831	0.78912353359253
C	3.24227982653280	1.24232954032831	0.78912353359253
H	-3.85355539436309	-0.74010696253683	-1.84558687374712
H	-3.47723818252820	0.99549735714776	-1.85873959787837
H	3.47723818252820	-0.99549735714776	-1.85873959787837
H	-3.59035160305153	-1.16144326755108	1.82132494998052
H	3.59035160305153	1.16144326755108	1.82132494998052
H	-3.81492662904010	-2.01400431162055	0.27810217123179

H	3.81492662904010	2.01400431162055	0.27810217123179
H	-4.93555547951086	1.60153049484224	-0.09909126926157
H	4.93555547951086	-1.60153049484224	-0.09909126926157
H	-4.94143364286320	0.74068315725493	1.45290579840549
H	4.94143364286320	-0.74068315725493	1.45290579840549
H	-6.64010901441005	0.02918602684251	0.03386614438771
H	6.64010901441005	-0.02918602684251	0.03386614438771
H	-2.51814082665376	1.10252167568866	1.64320030413504
H	2.51814082665376	-1.10252167568866	1.64320030413504
H	-2.54368679511682	1.95021248870836	0.26490527871508
H	2.54368679511682	-1.95021248870836	0.26490527871508
H	-1.72358810417854	-0.30653361094830	-2.65760215152762
H	1.72358810417854	0.30653361094830	-2.65760215152762
H	-1.74652396294314	-2.47810920022775	1.15510244256606
H	1.74652396294314	2.47810920022775	1.15510244256606
H	3.85355539436309	0.74010696253683	-1.84558687374712
H	0.49681184072091	0.59209320083116	3.13270365698378
H	-0.49681184072091	-0.59209320083116	3.13270365698378
H	0.85456359649022	-2.50596930090860	-1.71329785639944
H	0.61718255746135	2.67720172614348	-1.31901958299306
H	-0.85456359649022	2.50596930090860	-1.71329785639944
H	-0.61718255746135	-2.67720172614348	-1.31901958299306
TPSSH			
La	0.00000000000000	-0.00000000000000	-0.15989599976905
N	-2.33550767030419	1.02445318725673	0.59702629595119
N	2.33550767030419	-1.02445318725673	0.59702629595119
O	0.00000000000000	0.00000000000000	2.38702747077702
O	-0.23692419494220	2.23737046341116	-1.36361591740912
O	0.23692419494220	-2.23737046341116	-1.36361591740912
O	-1.78411037149192	-1.62896684861791	0.64421664804363
O	1.78411037149192	1.62896684861791	0.64421664804363
O	-1.98198974588399	-0.15407267904024	-1.77678193285212
O	1.98198974588399	0.15407267904024	-1.77678193285212
O	5.78776238941460	0.27224452553285	0.14058614591216
O	-5.78776238941460	-0.27224452553285	0.14058614591216
C	-3.36184705430022	0.02390401190320	-1.36629450461214
C	3.36184705430022	-0.02390401190320	-1.36629450461214
C	-3.40904979683633	0.09997372493478	0.15967462799510
C	3.40904979683633	-0.09997372493478	0.15967462799510
C	-4.77837576539878	0.63898273589404	0.59060197086426
C	4.77837576539878	-0.63898273589404	0.59060197086426
C	-3.16965958971230	-1.24310837400814	0.84458638026784
C	3.16965958971230	1.24310837400814	0.84458638026784
H	-3.96017542991861	-0.80862526600625	-1.72741250172328
H	-3.73426669679738	0.95560389717265	-1.79475674906296
H	3.73426669679738	-0.95560389717265	-1.79475674906296
H	-3.35271981530677	-1.14833739675984	1.91588501112394
H	3.35271981530677	1.14833739675984	1.91588501112394
H	-3.82095050252042	-2.00741853618450	0.42825657913263
H	3.82095050252042	2.00741853618450	0.42825657913263
H	-4.92187711363354	1.62880644460330	0.14869356148401
H	4.92187711363354	-1.62880644460330	0.14869356148401
H	-4.79611571398904	0.73312096548447	1.67997814410968
H	4.79611571398904	-0.73312096548447	1.67997814410968
H	-6.64582190715991	0.09015496135208	0.40899755665142
H	6.64582190715991	-0.09015496135208	0.40899755665142
H	-2.37575804388259	1.14120142333518	1.60890021501398
H	2.37575804388259	-1.14120142333518	1.60890021501398

H	-2.49928527989323	1.95105198199008	0.20466119196270
H	2.49928527989323	-1.95105198199008	0.20466119196270
H	-1.95793673061676	-0.35701184675204	-2.72466675860190
H	1.95793673061676	0.35701184675204	-2.72466675860190
H	-1.64607525341952	-2.50658240471313	1.03392460245136
H	1.64607525341952	2.50658240471313	1.03392460245136
H	3.96017542991861	0.80862526600625	-1.72741250172328
H	0.45702834780272	0.62584118769535	2.96949727351336
H	-0.45702834780272	-0.62584118769535	2.96949727351336
H	0.79483845385385	-2.29833323877567	-2.15588164776825
H	0.57489559117945	2.72399746062065	-1.57964392795147
H	-0.79483845385385	2.29833323877567	-2.15588164776825
H	-0.57489559117945	-2.72399746062065	-1.57964392795147

Supplementary Table 5. Cartesian coordinates for DOTA optimized structures (in Å)

PBE			
La	0.00000000000000	-0.00000000000000	-0.02208051359568
O	-1.50989245414785	1.79828018167765	0.79606193294926
O	1.50989245414785	-1.79828018167765	0.79606193294926
O	1.86550074746807	1.49083549000727	0.73573296989721
O	-1.86550074746807	-1.49083549000727	0.73573296989721
O	0.00000000000000	0.00000000000000	2.61085298985245
N	-1.88394890810143	0.97374007397341	-1.79786946577930
N	1.88394890810143	-0.97374007397341	-1.79786946577930
N	0.97325125120086	1.88768060347229	-1.81209028639549
N	-0.97325125120086	-1.88768060347229	-1.81209028639549
H	0.57399627186423	-0.52883439506167	3.19512304859344
H	-0.57399627186423	0.52883439506167	3.19512304859344
C	-2.72358937846553	1.92838497603165	0.36965283283524
C	2.72358937846553	-1.92838497603165	0.36965283283524
C	1.98747660635676	2.70845314695172	0.32534840322941
C	-1.98747660635676	-2.70845314695172	0.32534840322941
C	-3.07224243832932	1.22290392247482	-0.95271906494115
C	3.07224243832932	-1.22290392247482	-0.95271906494115
C	1.24241435093014	3.07283699506185	-0.96937036006987
C	-1.24241435093014	-3.07283699506185	-0.96937036006987
C	-1.43030076071749	2.26850604826626	-2.37465871012762
C	1.43030076071749	-2.26850604826626	-2.37465871012762
C	-2.25816140061636	-1.42531140854983	-2.40416786107716
C	2.25816140061636	1.42531140854983	-2.40416786107716
H	-3.52675497235640	0.25929259072624	-0.67911174677653
H	3.52675497235640	-0.25929259072624	-0.67911174677653
H	0.28642072571084	3.52574754601214	-0.66690922999238
H	-0.28642072571084	-3.52574754601214	-0.66690922999238
C	-2.20261409082443	0.01464544094247	-2.88673255922815
C	2.20261409082443	-0.01464544094247	-2.88673255922815
C	0.00096215146521	2.21792226746890	-2.88427989822664
C	-0.00096215146521	-2.21792226746890	-2.88427989822664
H	-1.52092003262302	3.03047116647084	-1.58969731503284
H	1.52092003262302	-3.03047116647084	-1.58969731503284
H	-3.03486676676196	-1.53185104965379	-1.63637196475861
H	3.03486676676196	1.53185104965379	-1.63637196475861
H	-1.43566733889382	0.11948018510381	-3.66480821997027
H	1.43566733889382	-0.11948018510381	-3.66480821997027
H	0.09459552939240	1.45988526928174	-3.67258508891603
H	-0.09459552939240	-1.45988526928174	-3.67258508891603

H	-3.83774476898930	1.80758183018221	-1.49274517051165
H	3.83774476898930	-1.80758183018221	-1.49274517051165
H	1.81084026388334	3.84214186820567	-1.52160252474311
H	-1.81084026388334	-3.84214186820567	-1.52160252474311
O	-3.63543631317588	2.55057885916532	0.96548975046819
O	3.63543631317588	-2.55057885916532	0.96548975046819
O	2.63312794601067	3.61110435109572	0.91283855292457
O	-2.63312794601067	-3.61110435109572	0.91283855292457
H	-2.09874460174937	2.57903841813293	-3.19876759503210
H	2.09874460174937	-2.57903841813293	-3.19876759503210
H	-2.55208336694084	-2.07788229924758	-3.24669480138755
H	2.55208336694084	2.07788229924758	-3.24669480138755
H	-3.16626291192907	0.27201700370653	-3.36372016038062
H	3.16626291192907	-0.27201700370653	-3.36372016038062
H	0.25075676229459	3.18692126229471	-3.35435070567863
H	-0.25075676229459	-3.18692126229471	-3.35435070567863
PBE0			
La	0.00000000000000	0.00000000000000	-0.03474063360853
O	-1.50150425178169	1.79454326812290	0.77436164478314
O	1.50150425178169	-1.79454326812290	0.77436164478314
O	1.85774598478483	1.48326344234305	0.71487216210653
O	-1.85774598478483	-1.48326344234305	0.71487216210653
O	-0.00000000000000	0.00000000000000	2.57475267445039
N	-1.87032686594454	0.96691961342046	-1.79282021638061
N	1.87032686594454	-0.96691961342046	-1.79282021638061
N	0.96689129828892	1.87550009773886	-1.80725389691353
N	-0.96689129828892	-1.87550009773886	-1.80725389691353
H	0.57632087768372	-0.51620396640197	3.14951528934170
H	-0.57632087768372	0.51620396640197	3.14951528934170
C	-2.70149343925011	1.91483190828437	0.35673982682392
C	2.70149343925011	-1.91483190828437	0.35673982682392
C	1.96854086650774	2.68813645890695	0.31542987688651
C	-1.96854086650774	-2.68813645890695	0.31542987688651
C	-3.04739838501422	1.21561016311421	-0.95618938817933
C	3.04739838501422	-1.21561016311421	-0.95618938817933
C	1.23502365556890	3.04920659001636	-0.97309357018680
C	-1.23502365556890	-3.04920659001636	-0.97309357018680
C	-1.42044286547341	2.24914450211610	-2.36449002450907
C	1.42044286547341	-2.24914450211610	-2.36449002450907
C	-2.23905065079794	-1.41590377102716	-2.39327941015409
C	2.23905065079794	1.41590377102716	-2.39327941015409
H	-3.50460332124527	0.26237719467903	-0.68270427610238
H	3.50460332124527	-0.26237719467903	-0.68270427610238
H	0.28933297542583	3.50662045667281	-0.67386661193078
H	-0.28933297542583	-3.50662045667281	-0.67386661193078
C	-2.18602535038463	0.01614469471141	-2.87096708041152
C	2.18602535038463	-0.01614469471141	-2.87096708041152
C	0.00309443430470	2.20228422255832	-2.86888035953875
C	-0.00309443430470	-2.20228422255832	-2.86888035953875
H	-1.51205375393391	3.00874500268643	-1.58809718384945
H	1.51205375393391	-3.00874500268643	-1.58809718384945
H	-3.01304868701582	-1.52268517386543	-1.63380552206945
H	3.01304868701582	1.52268517386543	-1.63380552206945
H	-1.42768623873665	0.12067412870905	-3.64634513112180
H	1.42768623873665	-0.12067412870905	-3.64634513112180
H	0.09729712599067	1.45356053229452	-3.65498033600221
H	-0.09729712599067	-1.45356053229452	-3.65498033600221
H	-3.80435521697719	1.80001214368230	-1.49121867632883

H	3.80435521697719	-1.80001214368230	-1.49121867632883
H	1.80600743194874	3.80899437927479	-1.51894994118485
H	-1.80600743194874	-3.80899437927479	-1.51894994118485
O	-3.60677583388016	2.52325750784224	0.94778983048679
O	3.60677583388016	-2.52325750784224	0.94778983048679
O	2.59464364865372	3.58528817598831	0.90274008738012
O	-2.59464364865372	-3.58528817598831	0.90274008738012
H	-2.08323075193354	2.55455552026209	-3.18390409641359
H	2.08323075193354	-2.55455552026209	-3.18390409641359
H	-2.52910060580269	-2.06238474012599	-3.23102788955162
H	2.52910060580269	2.06238474012599	-3.23102788955162
H	-3.14375200678807	0.27072290488288	-3.34182070064988
H	3.14375200678807	-0.27072290488288	-3.34182070064988
H	0.24916687120929	3.16583643223719	-3.33237942675108
H	-0.24916687120929	-3.16583643223719	-3.33237942675108
TPSSH			
La	0.00000000000000	-0.00000000000000	-0.03121247100158
O	-1.49015572135651	1.80949615955138	0.76128670982906
O	1.49015572135651	-1.80949615955138	0.76128670982906
O	1.87164407827416	1.46835473027340	0.70337241952392
O	-1.87164407827416	-1.46835473027340	0.70337241952392
O	-0.00000000000000	0.00000000000000	2.61647871598760
N	-1.86751281047378	0.96365698785569	-1.79470426113276
N	1.86751281047378	-0.96365698785569	-1.79470426113276
N	0.96236705951660	1.87137612999780	-1.80732607062925
N	-0.96236705951660	-1.87137612999780	-1.80732607062925
H	0.58569007805250	-0.50844779441012	3.19600136628660
H	-0.58569007805250	0.50844779441012	3.19600136628660
C	-2.70094508293772	1.92619321722573	0.34866289787697
C	2.70094508293772	-1.92619321722573	0.34866289787697
C	1.97725590249834	2.68490771130581	0.31115455427078
C	-1.97725590249834	-2.68490771130581	0.31115455427078
C	-3.05648930304839	1.20631072439169	-0.95490960091007
C	3.05648930304839	-1.20631072439169	-0.95490960091007
C	1.22573528587444	3.05638137307103	-0.96859376865174
C	-1.22573528587444	-3.05638137307103	-0.96859376865174
C	-1.42490130827980	2.25984440766768	-2.36811344430325
C	1.42490130827980	-2.25984440766768	-2.36811344430325
C	-2.24867846080785	-1.41956092243552	-2.39611476136770
C	2.24867846080785	1.41956092243552	-2.39611476136770
H	-3.49399629958689	0.24745833095514	-0.67228758547296
H	3.49399629958689	-0.24745833095514	-0.67228758547296
H	0.27521656548287	3.49584706143465	-0.66129311592083
H	-0.27521656548287	-3.49584706143465	-0.66129311592083
C	-2.18747483406954	0.01359911093551	-2.88577489613947
C	2.18747483406954	-0.01359911093551	-2.88577489613947
C	0.00027069308995	2.20477077293657	-2.88263381350036
C	-0.00027069308995	-2.20477077293657	-2.88263381350036
H	-1.50734644644735	3.01049977956035	-1.58309534836306
H	1.50734644644735	-3.01049977956035	-1.58309534836306
H	-3.01377209055071	-1.51624503135458	-1.62732687496388
H	3.01377209055071	1.51624503135458	-1.62732687496388
H	-1.42018190564061	0.11442688848448	-3.65203594050913
H	1.42018190564061	-0.11442688848448	-3.65203594050913
H	0.09119361670039	1.44748419930015	-3.66012483158724
H	-0.09119361670039	-1.44748419930015	-3.66012483158724
H	-3.81842364994005	1.77825703654352	-1.49440135896769
H	3.81842364994005	-1.77825703654352	-1.49440135896769

H	1.78594637399788	3.82012132115392	-1.51812117809350
H	-1.78594637399788	-3.82012132115392	-1.51812117809350
O	-3.60277167503255	2.54777001281944	0.94422257543565
O	3.60277167503255	-2.54777001281944	0.94422257543565
O	2.61752498909905	3.57774903805004	0.90258608520457
O	-2.61752498909905	-3.57774903805004	0.90258608520457
H	-2.09443475047749	2.56491831260265	-3.18055751224315
H	2.09443475047749	-2.56491831260265	-3.18055751224315
H	-2.53807947752052	-2.07440193094829	-3.22595030592659
H	2.53807947752052	2.07440193094829	-3.22595030592659
H	-3.14193349021338	0.27621749386635	-3.35650045391578
H	3.14193349021338	-0.27621749386635	-3.35650045391578
H	0.25617337075771	3.16522226369577	-3.34467510832213
H	-0.25617337075771	-3.16522226369577	-3.34467510832213

Supplementary Table 6. Cartesian coordinates for MACROPA optimized structures (in Å)

PBE			
La	0.00000000000000	0.00000000000000	-0.43970932993587
O	-1.86461343417776	-1.21367174977891	-2.14125051917647
O	0.71476279690183	-2.22907893806558	-1.78252438786090
O	1.86461343417776	1.21367174977891	-2.14125051917647
O	-0.71476279690183	2.22907893806558	-1.78252438786090
O	1.34383407829434	1.65722902967130	0.74601661258430
O	2.09302797409161	2.49283420111786	2.71230809126878
O	-1.34383407829434	-1.65722902967130	0.74601661258430
O	-2.09302797409161	-2.49283420111786	2.71230809126878
N	-2.79816572292281	0.88477218940007	-0.39230583810612
N	2.79816572292281	-0.88477218940007	-0.39230583810612
N	-1.06712683764056	1.23392945539238	1.67715103546364
N	1.06712683764056	-1.23392945539238	1.67715103546364
C	-3.65485025828112	0.19745413765285	-1.39070158945014
H	-4.71483547357436	0.21187957043045	-1.07839082501729
H	-3.59325372766378	0.74307069768297	-2.33929996143272
C	-3.22864626658369	-1.22567725075150	-1.63663525906133
H	-3.25516861715648	-1.83872857381785	-0.72323505407461
H	-3.88549837939642	-1.67795597433717	-2.39595688067097
C	-1.51324745795155	-2.54848834763928	-2.57243355382592
H	-2.13307365824470	-2.83226160065002	-3.43813785322036
H	-1.70000087811264	-3.25542218794192	-1.74701552736502
C	-0.06394762101978	-2.57314357767437	-2.95602293468160
H	0.19878426616587	-3.58854529162345	-3.28869033246692
H	0.16215427864198	-1.86286927885361	-3.76869508340041
C	2.07159484685773	-2.72478121008177	-1.89391806643617
H	2.52871630384241	-2.31347117685286	-2.80781653766038
H	2.04588768074098	-3.82141227687773	-1.98973263817558
C	2.82495036530501	-2.34846559905368	-0.64030110298370
H	2.35948686197603	-2.85085404333092	0.21683808086150
H	3.86327236872591	-2.72052704550616	-0.72413731391447
C	3.65485025828112	-0.19745413765285	-1.39070158945014
H	4.71483547357436	-0.21187957043045	-1.07839082501729
H	3.59325372766378	-0.74307069768297	-2.33929996143272
C	3.22864626658369	1.22567725075150	-1.63663525906133
H	3.25516861715648	1.83872857381785	-0.72323505407461
H	3.88549837939642	1.67795597433717	-2.39595688067097
C	1.51324745795155	2.54848834763928	-2.57243355382592
H	2.13307365824470	2.83226160065002	-3.43813785322036

H	1.70000087811264	3.25542218794192	-1.74701552736502
C	0.06394762101978	2.57314357767437	-2.95602293468160
H	-0.19878426616587	3.58854529162345	-3.28869033246692
H	-0.16215427864198	1.86286927885361	-3.76869508340041
C	-2.07159484685773	2.72478121008177	-1.89391806643617
H	-2.52871630384241	2.31347117685286	-2.80781653766038
H	-2.04588768074098	3.82141227687773	-1.98973263817558
C	-2.82495036530501	2.34846559905368	-0.64030110298370
H	-2.35948686197603	2.85085404333092	0.21683808086150
H	-3.86327236872591	2.72052704550616	-0.72413731391447
C	-3.30316912906246	0.60244723217325	0.97021682813383
H	-3.41051619257088	-0.48714294458917	1.07551489948425
H	-4.30078165828058	1.05374384043845	1.12647723745246
C	-2.35681816134512	1.09916389061813	2.03358007991785
C	-2.79339925310735	1.41997611013409	3.32409632292340
H	-3.84582276439846	1.30506937243789	3.58602359373130
C	-1.86845373646520	1.89443435272471	4.25273422118757
H	-2.18553916688146	2.14003647435413	5.26722321978700
C	-0.54123647704462	2.08634948564746	3.85953471540972
H	0.20828113217513	2.49671598916769	4.53532101059195
C	-0.18398443205251	1.75435823110350	2.55537636361722
C	1.20470906812856	1.99141339644503	1.98864348153423
C	3.30316912906246	-0.60244723217325	0.97021682813383
H	3.41051619257088	0.48714294458917	1.07551489948425
H	4.30078165828058	-1.05374384043845	1.12647723745246
C	2.35681816134512	-1.09916389061813	2.03358007991785
C	2.79339925310735	-1.41997611013409	3.32409632292340
H	3.84582276439846	-1.30506937243789	3.58602359373130
C	1.86845373646520	-1.89443435272471	4.25273422118757
H	2.18553916688146	-2.14003647435413	5.26722321978700
C	0.54123647704462	-2.08634948564746	3.85953471540972
H	-0.20828113217513	-2.49671598916769	4.53532101059195
C	0.18398443205251	-1.75435823110350	2.55537636361722
C	-1.20470906812856	-1.99141339644503	1.98864348153423
PBE0			
La	-0.00000000000000	0.00000000000000	-0.45099383625228
O	-1.84055422925754	-1.20082618390428	-2.12691056810674
O	0.71483943574183	-2.19315307573943	-1.79042413231572
O	1.84055422925754	1.20082618390428	-2.12691056810674
O	-0.71483943574183	2.19315307573943	-1.79042413231572
O	1.34487707577522	1.66370531938876	0.72990393745128
O	2.08684333354285	2.48933606081893	2.67318348209774
O	-1.34487707577522	-1.66370531938876	0.72990393745128
O	-2.08684333354285	-2.48933606081893	2.67318348209774
N	-2.75821083280721	0.87521078759410	-0.38377210066238
N	2.75821083280721	-0.87521078759410	-0.38377210066238
N	-1.04064683647907	1.22354659030242	1.66330549230230
N	1.04064683647907	-1.22354659030242	1.66330549230230
C	-3.60984559720295	0.20218307845863	-1.37567834739031
H	-4.66073735751617	0.21507168422722	-1.06242925964336
H	-3.55170772360926	0.74822022731507	-2.31508466078334
C	-3.18785710228803	-1.21118002776294	-1.63355757141551
H	-3.22292237696933	-1.82820728977386	-0.73318952411611
H	-3.84129426565297	-1.64932705103258	-2.39260284591438
C	-1.49276355351696	-2.50988291003533	-2.57667819280807
H	-2.10427180187426	-2.77729235261666	-3.44300882199170
H	-1.67988879647681	-3.22772415708410	-1.77190870304643
C	-0.04901565583661	-2.53033429852043	-2.95208836353666

H	0.21379849582678	-3.53587994180682	-3.28731415251364
H	0.17507066613498	-1.82239518625360	-3.75619608172993
C	2.04511170516933	-2.71101257464759	-1.86779891966062
H	2.52247401354667	-2.33276372850023	-2.77546071175066
H	2.00167888621637	-3.80063331889718	-1.94034670100678
C	2.78416585154530	-2.32661870707270	-0.61822276270756
H	2.31447969079943	-2.82062980230132	0.23150203167622
H	3.81512882128439	-2.69695171570741	-0.68982426937878
C	3.60984559720295	-0.20218307845863	-1.37567834739031
H	4.66073735751617	-0.21507168422722	-1.06242925964336
H	3.55170772360926	-0.74822022731507	-2.31508466078334
C	3.18785710228803	1.21118002776294	-1.63355757141551
H	3.22292237696933	1.82820728977386	-0.73318952411611
H	3.84129426565297	1.64932705103258	-2.39260284591438
C	1.49276355351696	2.50988291003533	-2.57667819280807
H	2.10427180187426	2.77729235261666	-3.44300882199170
H	1.67988879647681	3.22772415708410	-1.77190870304643
C	0.04901565583661	2.53033429852043	-2.95208836353666
H	-0.21379849582678	3.53587994180682	-3.28731415251364
H	-0.17507066613498	1.82239518625360	-3.75619608172993
C	-2.04511170516933	2.71101257464759	-1.86779891966062
H	-2.52247401354667	2.33276372850023	-2.77546071175066
H	-2.00167888621637	3.80063331889718	-1.94034670100678
C	-2.78416585154530	2.32661870707270	-0.61822276270756
H	-2.31447969079943	2.82062980230132	0.23150203167622
H	-3.81512882128439	2.69695171570741	-0.68982426937878
C	-3.25869165444257	0.58860859148010	0.96458804634109
H	-3.37650336490488	-0.49205720837062	1.06423177896027
H	-4.24354346426973	1.04659460746160	1.12392759955302
C	-2.31189504302733	1.06707937839320	2.02476288186232
C	-2.73512953001668	1.34899982037792	3.31837623521347
H	-3.77508490306532	1.21488972104991	3.58825755871487
C	-1.81075469228169	1.80837406760308	4.23980302092090
H	-2.11531999659806	2.02329200351225	5.25670452927611
C	-0.49889887114101	2.02405440144312	3.83846731959377
H	0.24892896110851	2.42330168230087	4.50979498521567
C	-0.15960222598162	1.72748154770365	2.53327249684577
C	1.21323075078747	1.98526261130107	1.95790871258002
C	3.25869165444257	-0.58860859148010	0.96458804634109
H	3.37650336490488	0.49205720837062	1.06423177896027
H	4.24354346426973	-1.04659460746160	1.12392759955302
C	2.31189504302733	-1.06707937839320	2.02476288186232
C	2.73512953001668	-1.34899982037792	3.31837623521347
H	3.77508490306532	-1.21488972104991	3.58825755871487
C	1.81075469228169	-1.80837406760308	4.23980302092090
H	2.11531999659806	-2.02329200351225	5.25670452927611
C	0.49889887114101	-2.02405440144312	3.83846731959377
H	-0.24892896110851	-2.42330168230087	4.50979498521567
C	0.15960222598162	-1.72748154770365	2.53327249684577
C	-1.21323075078747	-1.98526261130107	1.95790871258002
TPSSH			
La	0.00000000000000	0.00000000000000	-0.46201260457828
O	-1.82767105007788	-1.19043631689305	-2.13256556670513
O	0.72408509681675	-2.16753718561819	-1.81868517129781
O	1.82767105007788	1.19043631689305	-2.13256556670513
O	-0.72408509681675	2.16753718561819	-1.81868517129781
O	1.33620243799747	1.66589772288319	0.73457590238768
O	2.09207611223175	2.47097321873367	2.69398302580655

O	-1.33620243799747	-1.66589772288319	0.73457590238768
O	-2.09207611223175	-2.47097321873367	2.69398302580655
N	-2.74827306178214	0.87667342736790	-0.38006892670191
N	2.74827306178214	-0.87667342736790	-0.38006892670191
N	-1.04517134523862	1.20399874810441	1.67744828761629
N	1.04517134523862	-1.20399874810441	1.67744828761629
C	-3.60835787392920	0.21465663626691	-1.38787836501177
H	-4.65916382831860	0.23817341687948	-1.07788768756192
H	-3.53139694961538	0.76256306407554	-2.32373335131097
C	-3.19040957412749	-1.20720869281539	-1.63797468629836
H	-3.21857479265114	-1.81931472146472	-0.73554730461563
H	-3.83323372025191	-1.64874440517940	-2.40218008233311
C	-1.48500740935425	-2.50557781477543	-2.61368214473685
H	-2.10578784221163	-2.74606480823352	-3.47981188201227
H	-1.66731818225438	-3.23283259869541	-1.81827975706048
C	-0.03781663798799	-2.50791836043312	-2.99888371713076
H	0.24260741734432	-3.50613707505526	-3.33679529063340
H	0.18016873853975	-1.78508082217638	-3.78916885112096
C	2.05196167482965	-2.73247147233252	-1.86289910166836
H	2.55523424053522	-2.37855745304515	-2.76469312975264
H	1.96967514225213	-3.81970685831022	-1.91717221300824
C	2.77089165715791	-2.34224107252309	-0.59587245420458
H	2.27581203127116	-2.81452841978042	0.25046707438581
H	3.80048282348691	-2.71761965100614	-0.64219615820670
C	3.60835787392920	-0.21465663626691	-1.38787836501177
H	4.65916382831860	-0.23817341687948	-1.07788768756192
H	3.53139694961538	-0.76256306407554	-2.32373335131097
C	3.19040957412749	1.20720869281539	-1.63797468629836
H	3.21857479265114	1.81931472146472	-0.73554730461563
H	3.83323372025191	1.64874440517940	-2.40218008233311
C	1.48500740935425	2.50557781477543	-2.61368214473685
H	2.10578784221163	2.74606480823352	-3.47981188201227
H	1.66731818225438	3.23283259869541	-1.81827975706048
C	0.03781663798799	2.50791836043312	-2.99888371713076
H	-0.24260741734432	3.50613707505526	-3.33679529063340
H	-0.18016873853975	1.78508082217638	-3.78916885112096
C	-2.05196167482965	2.73247147233252	-1.86289910166836
H	-2.55523424053522	2.37855745304515	-2.76469312975264
H	-1.96967514225213	3.81970685831022	-1.91717221300824
C	-2.77089165715791	2.34224107252309	-0.59587245420458
H	-2.27581203127116	2.81452841978042	0.25046707438581
H	-3.80048282348691	2.71761965100614	-0.64219615820670
C	-3.26742494939290	0.57409603031666	0.97035193676732
H	-3.38100370674182	-0.50736921615778	1.05290207021864
H	-4.25222425608167	1.03249965264051	1.12095827544030
C	-2.32324581931654	1.04999309692199	2.03984270751756
C	-2.74887686286220	1.33614069205226	3.33541746882826
H	-3.78949906023685	1.20767673666510	3.60277330981088
C	-1.82226101963440	1.79391384858670	4.26248746305918
H	-2.12902338894439	2.01256969428631	5.27713426479231
C	-0.50553417984879	2.00631154368903	3.86297757826260
H	0.24077586559661	2.40354602828960	4.53625460831193
C	-0.16214817811105	1.70981956224428	2.55390721930168
C	1.20782007165137	1.97268016906433	1.97549745115401
C	3.26742494939290	-0.57409603031666	0.97035193676732
H	3.38100370674182	0.50736921615778	1.05290207021864
H	4.25222425608167	-1.03249965264051	1.12095827544030
C	2.32324581931654	-1.04999309692199	2.03984270751756

C	2.74887686286220	-1.33614069205226	3.33541746882826
H	3.78949906023685	-1.20767673666510	3.60277330981088
C	1.82226101963440	-1.79391384858670	4.26248746305918
H	2.12902338894439	-2.01256969428631	5.27713426479231
C	0.50553417984879	-2.00631154368903	3.86297757826260
H	-0.24077586559661	-2.40354602828960	4.53625460831193
C	0.16214817811105	-1.70981956224428	2.55390721930168
C	-1.20782007165137	-1.97268016906433	1.97549745115401

Supplementary Table 7. Cartesian coordinates for PSMA optimized structures (in Å)

PBE			
La	-3.19932224000119	-1.95609475155897	0.62779424842739
N	-1.32817816401482	-3.17551059576809	-0.99478957679604
N	-0.89098610672625	-2.61389752268679	1.92979192226813
N	-1.78435975300406	0.22706828449130	1.50027583333734
N	-2.19832410352395	-0.32664107687267	-1.41498860094948
C	-2.61379672915360	-3.44228422191541	3.55138325592965
C	-0.43694610052161	-1.46546847397365	2.76387662969786
O	-2.86378354571303	-4.07697839112438	4.60193721933929
C	-2.08481105338534	-4.16962265500966	-1.78918814700125
H	-1.40835427323924	-4.88288375182234	-2.29161034579492
H	-2.64736745762558	-3.64597365889099	-2.57577903761113
C	-0.62706219662564	-2.22009392144387	-1.89256773223859
H	-0.13580747998879	-2.75736472948897	-2.72409915632910
H	0.17629710086583	-1.74315454023330	-1.31742567343176
C	-1.55323422468045	-1.16125152378460	-2.46943368824406
H	-0.98232118163835	-0.52249268599066	-3.16703183092146
H	-2.34844179720069	-1.63853682579186	-3.05472857096430
O	-3.56757813218538	-6.01879419651555	-1.43570124763573
O	-4.50396424391872	0.02167777351150	1.23727224178080
H	-1.10759602293606	-1.40242384490001	3.63041879317387
H	0.57753329819760	-1.65495528524359	3.15828220143213
C	-1.28478686462635	-3.72342899280610	2.82773737963602
H	-0.49529314366744	-3.94319777681727	3.56717187003885
H	-1.43609060515744	-4.63505645559422	2.23045779443731
C	0.18725638321927	-3.04739254464908	1.00307587363872
H	0.96192931809871	-3.61790920151887	1.54636333951933
H	0.68234727325164	-2.14852808724780	0.61412702056689
C	-0.33903947471378	-3.89568496245553	-0.14450933840813
H	0.51230672408698	-4.24476330517865	-0.75682745898292
H	-0.83445123612561	-4.79302337728283	0.24851725730276
O	-3.39568748882322	-2.57814008936513	2.99211693186717
O	-3.50950072920848	-4.40463137029853	0.14714255956778
C	-3.12680983608317	-4.94529614026607	-0.96795771934863
H	0.24425228428536	-0.19619571471075	1.15582333899829
C	-3.38055808779455	0.34697961453362	-1.98432091503316
H	-3.14650853109715	0.83655992606337	-2.94659781763527
H	-3.71708614793140	1.13384206648856	-1.29430488615827
C	-1.23543658740889	0.68336672020350	-0.90107338847599
H	-1.10287687784589	1.50514159880619	-1.62755355714232
H	-0.25812958229027	0.19312301956634	-0.80800500237415
C	-1.65952461933451	1.26199254659970	0.43754445363201
H	-0.93916560396844	2.04345162031918	0.73994442062018
H	-2.63426758397171	1.75799043907066	0.34052924043310
O	-4.45618855969139	-1.79330026519516	-1.62877744768677
H	-0.04359634558059	0.64686471276592	2.67699045442374

C	-4.52216419662824	-0.64643315158029	-2.16228297164862
C	-2.64423727948359	0.76515041829309	2.57885538313034
H	-2.29027853124789	1.75254664291119	2.92271067253225
H	-2.61079570010362	0.08186676954896	3.44097703203144
C	-0.43916902717867	-0.14321727604558	2.01345750681203
C	-4.12227338417425	0.86229877537618	2.14885543759865
O	-4.85237402089496	1.70091162997571	2.71723861453661
H	-6.30074773306701	-1.01487291064636	-2.94749840109094
O	-20.02969386033362	-0.14766446686192	-6.64252422214207
O	-18.75665780102289	-1.30646825864582	-8.13167909345940
H	-19.61814483528147	-1.34548384216550	-8.60652560437294
N	-5.57444630322701	-0.29563497346734	-2.90832067849307
C	-6.02944067246680	1.06127044057024	-3.24875984714844
H	-6.40069324193373	1.04279789025220	-4.28322443674962
H	-5.17952054502557	1.75452314959014	-3.21024182413856
C	-7.14361711567460	1.47238988456777	-2.27614618561242
C	-6.62174685033488	1.78216894282191	-0.87197094555711
C	-7.99157144968304	2.65992246978290	-2.77366388275688
C	-7.78901462886815	1.83362964009723	0.13238782337374
C	-8.90759461306374	3.22248533603272	-1.64602858060467
C	-9.13374279504471	2.14738356961674	-0.58005518855207
H	-7.80758713127722	0.59210820441788	-2.20782524420769
H	-5.89821952230820	1.03604224391671	-0.51636257491052
H	-6.09324032718402	2.74993726956284	-0.90407883971487
H	-8.60137830832154	2.33419453523813	-3.63016785960434
H	-7.32659039126503	3.45921527862308	-3.13886820677845
H	-7.86687022438293	0.87720576128721	0.66751025529750
H	-7.59280876679666	2.60025546934778	0.89626333668304
H	-9.86341036117960	3.56324009403993	-2.06805049468579
H	-8.43795620780545	4.09815574787364	-1.17226940148384
H	-9.47694822374589	1.23077884792032	-1.08782104504001
C	-10.17248698248668	2.45516878237679	0.48890395452861
O	-10.49508195850153	3.59482008196472	0.86275975613271
N	-10.68457014653850	1.30823944143862	1.04638736870588
H	-10.22408586534692	0.45105084038102	0.73744918312290
C	-11.32576465084974	1.20318845868321	2.35695922580188
H	-11.47943416813594	2.22373926701187	2.73803582046260
C	-10.34579272355678	0.42188185206038	3.26546870947541
H	-8.95694897473451	2.17373149302663	3.04342933693062
H	-10.18195057658122	-0.56085702257770	2.77898448347601
C	-9.00932544969955	1.11997363838959	3.32969810037151
C	-7.89192671508769	0.45987392673408	3.69161134299146
C	-10.75685884430739	0.14425083626323	4.68645348654662
C	-7.84842421560550	-0.91113430207236	4.18670904719908
C	-10.08215138616681	-0.77163286319999	5.41610744234468
C	-8.87586179861754	-1.48977317246999	5.00163340005894
C	-6.66958922160006	-1.65609474250513	3.95010063849021
C	-8.65181203956781	-2.77964654192179	5.53998272897938
C	-6.49874732753986	-2.93882850407746	4.45057553093541
C	-7.50040773168587	-3.50523777083772	5.25675724068274
H	-11.60903051138071	0.67672718169352	5.11013028342556
H	-6.92546846813533	0.96199906191770	3.57510824690848
H	-10.45912454835486	-1.01891081195828	6.41380783771902
H	-5.88232397566714	-1.20939743909099	3.33922546494100
H	-5.57734642386744	-3.47968801307187	4.22577701070756
H	-7.37149427659989	-4.50703722365534	5.67222115328770
H	-9.41779080966007	-3.21027311984506	6.18991656667423
C	-12.71003717729204	0.52299804297613	2.27794115749977

O	-13.38713425030396	0.33768513920311	3.31027058240734
N	-13.09142331631489	0.15494692693241	1.04037183265583
H	-12.46082728167222	0.40898757159528	0.28041804548097
C	-14.30322778955063	-0.57960581873838	0.70060128872662
C	-13.96768809945761	-1.98057441532826	0.16938308753002
C	-15.14709869788060	-2.67855776213748	-0.52032810260022
C	-15.47533421363101	-2.04544469704496	-1.87438209498212
H	-14.85419795155168	0.00316723792447	-0.05241850995250
H	-14.92423173592744	-0.63783738302989	1.60336572635723
H	-13.13546236328954	-1.89608271308475	-0.55018142827996
H	-13.59998784210303	-2.59514964055801	1.00502633804803
H	-16.03858418584907	-2.65448093707401	0.12880430311981
H	-14.89086392259932	-3.73926121775479	-0.65904874199714
H	-14.58035820394131	-2.05666057749557	-2.51826293899810
H	-15.76406504927739	-0.99178271607412	-1.74548968645581
C	-16.64385017632494	-2.70344448168841	-2.64699187846036
H	-17.53487612040731	-2.73675809790124	-2.00933260197304
C	-16.29763820596931	-4.14180872721621	-3.00713371455969
O	-16.50739476768212	-5.10285877322910	-2.27833941413288
O	-15.66714256766857	-4.23553058214407	-4.20304482079163
H	-15.43957686403333	-5.18342055452007	-4.33894237639789
O	-18.51107326725144	-0.63208509525087	-2.71949692291416
H	-16.23428149334283	-1.87652892602547	-4.53927889490426
N	-16.98277993632282	-1.94109981175706	-3.84532334388754
C	-17.83778196276279	-0.87055135498839	-3.74474419444372
N	-17.90876023450897	-0.03594059179632	-4.84959487962071
H	-18.68769534805503	0.61912361144478	-4.76109871948877
C	-15.32844653270399	0.71417434601101	-6.38826947448931
H	-15.37323956883660	0.85535889367211	-5.29926043114494
C	-14.48795533149462	-0.49375090302079	-6.71438039891139
O	-13.78151901355401	-0.62730990683363	-7.70499599356451
O	-14.61722342829369	-1.47974558147987	-5.77639021302242
H	-14.08996987583711	-2.25119524682779	-6.08703729392264
C	-16.75389490689984	0.59750745762199	-6.96223403712893
C	-17.63822000555341	-0.43483514304254	-6.22509639850057
C	-18.95153691972921	-0.60569628255899	-6.99342327876257
H	-14.83790732133577	1.58567640077408	-6.83750974148142
H	-17.23463314413553	1.58333368739108	-6.88434126152448
H	-17.14419247705644	-1.41428595869654	-6.23813384097858
H	-16.70827612859421	0.33827997746463	-8.02892168343567
H	-5.37166313622117	-3.82702715642854	0.53186522275168
O	-5.67762672312503	-2.96566013989366	0.90467010905175
H	-6.29162029298026	-2.61451995231919	0.22985658303019
PBE0			
La	-3.29171451908754	-2.04864944834886	0.49946732808164
N	-1.38211909865714	-3.19058855533131	-1.07062844497016
N	-1.02612337274041	-2.63888796617015	1.84706735179273
N	-1.96750808096858	0.16393566497297	1.41354751037155
N	-2.31138317645765	-0.38167961591644	-1.48875937711254
C	-2.75687990292942	-3.52570695556403	3.40020658094403
C	-0.62512318171012	-1.49453237030637	2.68762058595353
O	-3.02017508597916	-4.18620787642798	4.41414866738593
C	-2.09623891251919	-4.19539758236194	-1.86475548068119
H	-1.40135381384394	-4.88990466008278	-2.34930506149388
H	-2.65906530642940	-3.69740583979872	-2.65697652688681
C	-0.70215034948345	-2.22422472804524	-1.94881933022908
H	-0.19565212460136	-2.74203306548525	-2.77243628033449
H	0.08099208941522	-1.73434978412229	-1.37133880998027

C	-1.64188760273764	-1.19122618853790	-2.52713234181852
H	-1.08230878536666	-0.54135659822554	-3.21056842648624
H	-2.41317576365782	-1.68161596360744	-3.11923495856840
O	-3.50685925112915	-6.07160968044552	-1.49914279123053
O	-4.67245630881491	-0.11757807338473	1.17760792550657
H	-1.31217258550602	-1.44879113814948	3.53246486371530
H	0.37581673741626	-1.66118751338372	3.10333344833263
C	-1.40643202947045	-3.75188684352495	2.72314035120974
H	-0.64107472287954	-3.94288254088773	3.48311704917974
H	-1.50713871648694	-4.66321108849673	2.12960417563246
C	0.07235634833090	-3.03162268380242	0.94852143549079
H	0.84439727615278	-3.58120467927267	1.50038703503979
H	0.54936260780831	-2.12410024916904	0.57943603714189
C	-0.39952539962461	-3.87888863036788	-0.21112322473052
H	0.46915465990930	-4.19277102934337	-0.80273163272977
H	-0.86805905832177	-4.78954909666758	0.16241048205636
O	-3.53671520927132	-2.68305080820485	2.84145063297380
O	-3.53057563283852	-4.44522076497626	0.03249095043117
C	-3.11740612104725	-4.98493657617702	-1.04988837038028
H	0.06153494986776	-0.21144091839934	1.11241578513900
C	-3.48039839009728	0.27364778316056	-2.07029495333741
H	-3.24313466162032	0.74476598583958	-3.03163019696045
H	-3.82344482693261	1.06703917428050	-1.40426224145252
C	-1.38388812048458	0.63186646714053	-0.95734543653335
H	-1.25459399839532	1.45252101098536	-1.67284136363937
H	-0.40559562051816	0.16604690091401	-0.84752527520171
C	-1.84269198919515	1.19350104120716	0.36621232376316
H	-1.14880518551206	1.98140536787565	0.68322665971974
H	-2.81602856193864	1.67167816864657	0.25009095373165
O	-4.52948939512062	-1.85860745523464	-1.75047439788534
H	-0.27794965595310	0.61171652798339	2.61732432538390
C	-4.61475380479152	-0.71102536688287	-2.24501244094248
C	-2.83822796610951	0.68555232685138	2.47148575700754
H	-2.50275780786918	1.66953326681436	2.81628983304364
H	-2.81137724522334	0.00790728735425	3.32846111066929
C	-0.63859152358383	-0.17791996515254	1.94682223623633
C	-4.29941077690089	0.76551740329694	2.03257696344930
O	-5.02292062925849	1.62386896908951	2.54040570823173
H	-6.40125341700814	-1.05279793061991	-2.98043452189500
O	-19.78530339489258	-0.47505792095887	-6.83884759710794
O	-18.40560917239154	-1.60888601083758	-8.20119077640450
H	-19.23076317593482	-1.74158706844612	-8.69770943972840
N	-5.68194522356288	-0.34094229990832	-2.93320655378440
C	-6.12713372690085	1.01575649973307	-3.23238725391279
H	-6.52030470913611	1.02524864008605	-4.25003054349078
H	-5.27551106458028	1.69546439858271	-3.20267564154935
C	-7.20193686208782	1.41806671130436	-2.22956716384545
C	-6.64234595891897	1.67422865894306	-0.84095809314973
C	-8.02550769188867	2.63331764316713	-2.66419026808181
C	-7.77359816445875	1.70046272121632	0.18640295316311
C	-8.86108078599099	3.19985259882354	-1.49343590755000
C	-9.10953870168460	2.10329390638394	-0.47024926204129
H	-7.87930783014392	0.55758185444964	-2.16872038550760
H	-5.92271425091915	0.91648124683758	-0.53273847103481
H	-6.10957605496693	2.63126107829543	-0.85304042992121
H	-8.68113628695613	2.34759112551133	-3.49064216762594
H	-7.35644531696411	3.41166528492148	-3.04367614748037
H	-7.87298978450191	0.71987715378988	0.65584604154220

H	-7.52984370617194	2.39950923205934	0.98866505494673
H	-9.80211484685018	3.60886563286934	-1.86474308223902
H	-8.32794890820326	4.02151095851255	-1.00826625719063
H	-9.51119331437264	1.23271394284920	-0.99840248822978
C	-10.09345422581011	2.41681787935934	0.63246712767674
O	-10.35889461831238	3.54452962725237	1.03951513898132
N	-10.62379710751347	1.28714306733559	1.17254565390583
H	-10.21435598548322	0.42836409536505	0.82998537665527
C	-11.25013889495261	1.17603012940790	2.47484144854993
H	-11.38732290996088	2.18557179863991	2.86872382706820
C	-10.28864149378990	0.37636193794320	3.36645390106784
H	-8.88426618596072	2.09384113591122	3.12234915569065
H	-10.14107571410033	-0.59817070155575	2.87734060741641
C	-8.95106908864510	1.05389623832601	3.42585156081373
C	-7.85104733032148	0.40086714341663	3.80514772072341
C	-10.70366771318559	0.09960191470832	4.77816257207718
C	-7.81682165566744	-0.96433760100070	4.30484917696517
C	-10.04467569955460	-0.80986581289031	5.50546335180010
C	-8.84640975325249	-1.53609841902409	5.09375509571515
C	-6.64679971876893	-1.70746106157838	4.08218974190767
C	-8.64643961262078	-2.82555714476157	5.61325901983567
C	-6.49311049798197	-2.98924917967214	4.56420247010657
C	-7.50682459349593	-3.55392025067732	5.33859891373498
H	-11.54866045113119	0.63072817191308	5.19723641052397
H	-6.89108285193996	0.89666374707815	3.69718476500903
H	-10.41798952088669	-1.04773246449461	6.49786599257005
H	-5.84503916909636	-1.25659111749879	3.50794083316293
H	-5.57722617583237	-3.53006792442802	4.35281686152836
H	-7.39485458102578	-4.55528209812363	5.73871358543998
H	-9.41930872818527	-3.25364366549734	6.24307423490929
C	-12.63599077632679	0.52596363566362	2.38736264029188
O	-13.27607230284599	0.26777495176038	3.41057982676857
N	-13.06455680854616	0.27802135682076	1.15023954051881
H	-12.44877344357675	0.55443181596151	0.39970590094847
C	-14.26880620902885	-0.44239391275764	0.80193030921876
C	-13.93454003353387	-1.82599340864317	0.25581228209386
C	-15.09443125139825	-2.50295108887757	-0.46304011088007
C	-15.40545470501279	-1.84621457305227	-1.79971425566762
H	-14.81261186334150	0.14783975234438	0.06213143813325
H	-14.88972413570371	-0.51312594323506	1.69426645293162
H	-13.09467492185994	-1.73380026840779	-0.44133584465701
H	-13.58762773126619	-2.45299752254283	1.08093537657373
H	-15.98830172644026	-2.49997061724088	0.16966590423466
H	-14.83217124009203	-3.55227193942101	-0.62074657817565
H	-14.49532994111703	-1.79586550820310	-2.40631642050527
H	-15.75067498489728	-0.82012403146169	-1.65646723437816
C	-16.49522887887495	-2.54656893642504	-2.61925745330387
H	-17.41770728480117	-2.59983338833024	-2.04579227208627
C	-16.08416867358041	-3.96898047012311	-2.93627324925325
O	-16.44698587729888	-4.94267718243909	-2.31538894019905
O	-15.22550849009983	-4.02471842735941	-3.95682642162964
H	-14.98016541302784	-4.95455113600012	-4.09758905767025
O	-18.43735885292424	-0.59448969567642	-2.90680620810858
H	-15.99598122430566	-1.75812092348976	-4.49281951797195
N	-16.77416249687414	-1.83685440115547	-3.85006527036961
C	-17.70004301603644	-0.84180276982828	-3.86650841024099
N	-17.76457434740199	-0.09220461775209	-5.01415609141115
H	-18.56234966172975	0.52971351460501	-4.99786579156866

C	-15.20850098722199	0.72613276781344	-6.50253423386762
H	-15.30605291616660	0.94520164543577	-5.43851421654141
C	-14.28375494801042	-0.43703234074278	-6.69604888187230
O	-13.54118076118366	-0.59495198032568	-7.63938230510029
O	-14.37952451337918	-1.33986391174531	-5.70312278309619
H	-13.79355587532695	-2.08593503885378	-5.91697933151324
C	-16.58633703658671	0.46614615549094	-7.11307181170578
C	-17.41973301604319	-0.56168145820436	-6.33610193240079
C	-18.67951645963607	-0.87031091672294	-7.12787279960593
H	-14.75836133682515	1.58424220304323	-6.99834299621688
H	-17.13378576358678	1.41035738021600	-7.13152625419510
H	-16.86919914467935	-1.49861370254800	-6.26557584238910
H	-16.47877926160765	0.13410590293125	-8.14644934265379
H	-6.30438846405254	-2.58195713446969	-0.08069010670413
O	-5.91011493550305	-2.50174241132255	0.79851496704568
H	-6.07678605812127	-1.58082897390551	1.06513148143402
TPSSH			
La	-2.57211810964194	0.15854327451099	-0.93763466369912
N	-0.86201678231031	-0.83331548236388	-2.81444246506190
N	-1.01187466354177	2.05742620705108	-2.06657828151376
N	-3.97148482992623	1.98462326882966	-2.40346513947643
N	-3.82715557331900	-0.89213099122796	-3.13998238064038
C	-0.78081613623931	2.64486938360475	0.34309843926183
C	-1.82680692377827	3.24885876947433	-2.42437342384986
O	-0.22737377542236	3.34312847089276	1.21141777383353
C	-0.28017970648836	-2.05266296828576	-2.21746415953306
H	0.58164325625816	-2.40487168818985	-2.79270980895302
H	-1.02983698659332	-2.84567818784195	-2.22613902500014
C	-1.53726208941992	-1.16801998193238	-4.09180870980463
H	-0.91895798824657	-1.85072718615979	-4.68481972355111
H	-1.63492328415464	-0.25100548311838	-4.67019841763426
C	-2.89872296234959	-1.79852754338877	-3.86885838721244
H	-3.33056409448046	-2.08036098483056	-4.83520356511759
H	-2.79852433312241	-2.70806616530705	-3.27928681132252
O	0.93608455078198	-2.64871353881949	-0.25337916037509
O	-4.86384004706309	0.63142300826122	-0.23150119069602
H	-2.08543469603363	3.75629574097503	-1.49565374952145
H	-1.23227498100682	3.94826210758516	-3.02249363974216
C	-0.06933486688127	2.44531813392476	-0.99710036878974
H	0.48391564723404	3.35154332306695	-1.26180969502516
H	0.65556241215995	1.64287068833372	-0.85073776513660
C	-0.27605610293481	1.55724430757371	-3.25325967344517
H	0.56875872343336	2.21458365086069	-3.48624988202303
H	-0.95106797193650	1.59682657868684	-4.10684893250167
C	0.23460621456048	0.14265881374974	-3.05327985790106
H	0.82374707625972	-0.15683896219224	-3.92677237887120
H	0.89713126072876	0.10518625160114	-2.18962356570733
O	-1.90613390495579	2.03797134202932	0.48934703373431
O	-0.44523299508296	-0.88204052969081	-0.12994786695176
C	0.12404401477403	-1.85419294254397	-0.75679295392057
H	-2.83804700270808	2.38177628588975	-4.11373547632469
C	-4.95788057194458	-1.67985414840275	-2.62176436398410
H	-5.35030253094935	-2.35766248217103	-3.38711525478289
H	-5.76705020119545	-1.00584680007136	-2.34157321795592
C	-4.33397917802578	0.15806057020057	-4.05956995071586
H	-5.07571794088533	-0.25941250568345	-4.74864471698208
H	-3.49629351489432	0.49880231291549	-4.66486843173643
C	-4.94445229111200	1.32232540970238	-3.30718003139797

H	-5.35043485696181	2.04410422297474	-4.02489631431886
H	-5.77776676745480	0.97045102168400	-2.70194010731403
O	-3.42490311833411	-2.25331567642580	-0.85298011504629
H	-3.62135877777730	3.80526569320062	-3.45379611697495
C	-4.54811668875110	-2.45996068357005	-1.38765633458993
C	-4.72939496211707	2.73257147671875	-1.38055621051414
H	-5.47297010975463	3.39462457714016	-1.83593336391335
H	-4.03870198763007	3.35183804793613	-0.80499024650000
C	-3.08938432277349	2.88687555799107	-3.18198363307836
C	-5.41383153101073	1.78533926960306	-0.38753228140454
O	-6.42145812495695	2.18676494611194	0.21828892595607
H	-5.07154516302873	-3.81667514607405	-0.04379396852937
O	-10.56278628783963	5.11569162107444	-4.28607737387476
O	-9.22671567605243	3.76526679311454	-5.50634010165403
H	-9.10005121715528	4.58944197673597	-6.01471292925833
N	-5.39378186368237	-3.34635806507445	-0.88266393663259
C	-6.79401736039890	-3.55075868049992	-1.25929823413910
H	-6.92798266426386	-4.59650989050728	-1.53945791125225
H	-7.01154148381195	-2.93888751562256	-2.13265292922074
C	-7.73936290691837	-3.18288334538974	-0.11296783191431
C	-7.62868053586196	-1.71901129158846	0.31074250832929
C	-9.19309287080393	-3.50110530687325	-0.52439806919012
C	-8.43995005447780	-1.46147525945787	1.58952485250096
C	-10.21393880749982	-2.61510102717583	0.20929350341545
C	-9.73054340760449	-2.30700794709542	1.63974952541588
H	-7.47171138406750	-3.80687514841840	0.74712687578723
H	-6.59133784138917	-1.42487669702894	0.47183448003708
H	-8.00001067145671	-1.09099784361090	-0.50276810474387
H	-9.40473873268776	-4.55330646749065	-0.32471675507737
H	-9.31073615919444	-3.35238749870631	-1.60225726091777
H	-7.83208342218838	-1.69194116030021	2.46698247156185
H	-8.71019132304570	-0.40600902714771	1.64534109954183
H	-11.18386871614308	-3.11579417665702	0.22883304370751
H	-10.35329517399590	-1.66807949680163	-0.31794740784933
H	-9.53501278141687	-3.25836927581686	2.14139999342417
C	-10.83336399485016	-1.58817905893065	2.38865991750076
O	-11.08508760452803	-0.38690003161389	2.22698760567026
N	-11.60610982119706	-2.35474491625195	3.19640129684381
H	-11.33569805229267	-3.31206590076881	3.37604619648880
C	-12.72615941971238	-1.78296348275778	3.93051346794488
H	-12.37996296383642	-0.94168599672740	4.53384510555961
C	-13.32562679442836	-2.86394299554956	4.83880067052525
H	-11.40321421737729	-2.80167756583787	5.98581631358217
H	-13.62091453119111	-3.71237970051065	4.20773728439885
C	-12.32671857239897	-3.35685753714101	5.85552200835550
C	-12.55648675972265	-4.45982452342198	6.58127755091003
C	-14.54733861744734	-2.39577036427113	5.58315066338117
C	-13.78355311747514	-5.25155124387848	6.57207745944598
C	-15.33188423523596	-3.26315942108774	6.23698162431104
C	-15.08045099540470	-4.69211626067261	6.40761010868513
C	-13.67580443746567	-6.62496574883648	6.86340832722719
C	-16.19781649882706	-5.53896985332155	6.53820270110334
C	-14.78745885140000	-7.44474672415388	6.93990791967857
C	-16.06324140754625	-6.89536857144622	6.77445316759839
H	-14.79659467744781	-1.34097152226328	5.56225045879798
H	-11.77076357863807	-4.81960485507795	7.23891491023798
H	-16.25038402967140	-2.89375225689741	6.68345272379799
H	-12.68733748668695	-7.04065300111336	7.02412437968714

H	-14.66941444380844	-8.50209889930297	7.14401249337394
H	-16.94279298042587	-7.52350977779838	6.84783734274602
H	-17.18652878084761	-5.10371928333029	6.44423977714294
C	-13.77823206764076	-1.19996236461320	2.97722111931344
O	-14.39252839802520	-0.16298054326199	3.26654747914844
N	-14.00319061418485	-1.89955347425435	1.85713001433525
H	-13.37828869506729	-2.67070592260659	1.66403130966530
C	-14.78275163461948	-1.36141350963411	0.74328921838013
C	-14.64913056865546	-2.26194234140480	-0.48454663538016
C	-14.85638549191532	-1.49170458674571	-1.79305756221532
C	-13.65177725501498	-0.60146712374892	-2.09557164870705
H	-14.41330824706528	-0.35461249030700	0.53340298617347
H	-15.82761601986850	-1.26673659986331	1.04204287118104
H	-13.64690700308662	-2.70382248331916	-0.50323447296688
H	-15.35800437745225	-3.08826430538324	-0.41220640107865
H	-15.76539217778085	-0.88545984760335	-1.73231910229696
H	-15.00461033588469	-2.20524459122174	-2.60606085242307
H	-12.75631107363734	-1.22012300094802	-2.20171618903318
H	-13.47088697102223	0.08662645278327	-1.26709753348254
C	-13.78718634021338	0.28557096566529	-3.34864795164158
H	-14.66559981912420	0.91870245142998	-3.26346602402360
C	-13.96600321656796	-0.56751437689452	-4.58932185530777
O	-15.04656354790326	-0.86782952970872	-5.06065029268276
O	-12.79688932261752	-1.01275872030085	-5.08337338294081
H	-12.99263388313637	-1.57986702122866	-5.85357931931574
O	-13.65720824887027	2.87229721507703	-2.38088570316210
H	-11.73888831188419	0.66222119566131	-3.53740215000325
N	-12.62462116774083	1.15551607906488	-3.49331583611257
C	-12.62796453754070	2.36764925605579	-2.85856298501496
N	-11.41939216546705	3.01128887163030	-2.74965574433037
H	-11.53097752305307	3.96580988311603	-2.42788271251330
C	-9.04443762638424	1.26330516422351	-1.95258353641256
H	-9.98081477964913	1.21588088047225	-1.39657194847224
C	-8.90537290304447	0.06270895878747	-2.84642130072777
O	-7.85541814279547	-0.40651506449073	-3.23899927011089
O	-10.10148215994455	-0.45834243934638	-3.21519615338180
H	-9.93412647325733	-1.18540161326001	-3.84623372468496
C	-8.98479991815575	2.56710602803087	-2.76719358740381
C	-10.26053044585991	2.80669184586973	-3.60094547416600
C	-10.05934413594529	4.02890817049022	-4.48899013402472
H	-8.21337188277657	1.25766223617662	-1.24856315911350
H	-8.86412239602642	3.39802520646803	-2.07113395007366
H	-10.43141939127491	1.96175668655545	-4.26541458241490
H	-8.11813243269788	2.55595779107500	-3.42822242843106
H	-1.61313591844120	-1.11701531017823	1.41932125971878
O	-2.54700528369022	-0.85481581160253	1.56367666754064
H	-3.03836659219254	-1.69099567819434	1.50089030185897

Supplementary Table 8. List of calculated LFDFT parameters (in eV) that correspond to the La L_2-edge RIXS of La^{3+} in free ion (Atom) as well as La^{3+} in 9H2O, 1TRIS, 2TRIS, MACROPA, DOTA and PSMA: Configuration-average energy (ΔE), Slater-Condon integrals (F^k and G^k), and spin-orbit coupling constants (ζ). The calculations are done using DFT with the PBE0[6] functionals based on the optimized structures obtained with the same functional. The extraction of the parameters from the DFT calculations can be seen elsewhere.[7]

	Atom	9H2O	1TRIS	2TRIS	MACROPA	DOTA	PSMA	Ref.[a]
$\Delta E(4f^0)$	0	0	0	0	0	0	0	
$\Delta E(2p^5 4f^1)$	5644.61	5643.97	5644.03	5644.00	5643.97	5643.92	5643.91	
$\Delta E(2p^5 5d^1)$	5652.57	5649.62	5649.04	5649.13	5648.84	5648.81	5648.78	
$\Delta E(3d^9 4f^1)$	835.42	834.82	834.78	834.76	834.69	834.66	834.65	
$\Delta E(3d^9 5d^1)$	844.73	840.94	840.50	840.27	839.71	839.54	839.48	
$F^2(2p,4f)$	1.2088	1.1523	1.1528	1.1479	1.0935	1.1256	1.1210	1.305
$G^2(2p,4f)$	0.0928	0.0882	0.0882	0.0878	0.0836	0.0860	0.0857	0.116
$G^4(2p,4f)$	0.0600	0.0570	0.0570	0.0568	0.0541	0.0556	0.0554	0.075
$F^2(2p,5d)$	0.5428	0.5027	0.4485	0.3760	0.3272	0.3607	0.3408	
$G^1(2p,5d)$	0.4676	0.4256	0.3805	0.3184	0.2808	0.3056	0.2887	
$G^3(2p,5d)$	0.2765	0.2516	0.2249	0.1888	0.1662	0.1807	0.1707	
$F^2(3d,4f)$	6.5959	6.2938	6.2968	6.2706	5.9740	6.1499	6.1249	7.063
$F^4(3d,4f)$	2.9702	2.8264	2.8279	2.8156	2.6818	2.7601	2.7489	3.167
$G^1(3d,4f)$	4.4378	4.2195	4.2216	4.2029	4.0028	4.1193	4.1025	4.723
$G^3(3d,4f)$	2.6083	2.4797	2.4810	2.4700	2.3523	2.4208	2.4109	2.761
$G^5(3d,4f)$	1.8037	1.7147	1.7156	1.7080	1.6266	1.6740	1.6671	1.905
$F^2(3d,5d)$	1.0608	1.0277	0.9131	0.7626	0.6452	0.7368	0.6963	
$F^4(3d,5d)$	0.4407	0.4036	0.3607	0.3028	0.2675	0.2894	0.2735	
$G^0(3d,5d)$	0.2719	0.2492	0.2228	0.1870	0.1640	0.1788	0.1690	
$G^2(3d,5d)$	0.3576	0.3265	0.2919	0.2451	0.2180	0.2342	0.2213	
$G^4(3d,5d)$	0.2831	0.2583	0.2309	0.1939	0.1700	0.1852	0.1750	
$\zeta(2p)$	263.93	263.93	263.93	263.93	263.93	263.92	263.92	281.48
$\zeta(3d)$	6.8882	6.8880	6.8876	6.8880	6.8864	6.8825	6.8823	6.799
$\zeta(4f)$	0.0941	0.0896	0.0896	0.0892	0.0850	0.0875	0.0871	0.091
$\zeta(5d)$	0.1205	0.1097	0.0980	0.0823	0.0830	0.0788	0.0703	-

[a]taken from ref. [8]

Supplementary Table 9. Structure of the input file for the FDMNES calculations

! Fdmnes indata file
Filout
LaCALC/outputfile
Range
-80 0.1 10. 0.2 20. 1. 50. 5 150
Quadrupole
SCF
Relativiste
Spinorbite
Density
state˙all
Edge
L2
Z_absorber
57
Radius
6.5
Convolution
Gamma_max
5
Gamma_hole
1.5
Ecent
18.29
Elarg
13
Gaussian
0.25
Molecule
List of cartesian coordinates see also Supplementary Tables 2-7
End

Supplementary References

[1] Martina Benešová, Martin Schäfer, Ulrike Bauder-Wüst, Ali Afshar-Oromieh, Clemens Kratochwil, Walter Mier, Uwe Haberkorn, Klaus Kopka, and Matthias Eder. Preclinical evaluation of a tailor-made dota-conjugated psma inhibitor with optimized linker moiety for imaging and endoradiotherapy of prostate cancer. *Journal of Nuclear Medicine*, 56(6):914–920, April 2015.

[2] Nikki A. Thiele, Victoria Brown, James M. Kelly, Alejandro Amor-Coarasa, Una Jermilova, Samantha N. MacMillan, Anastasia Nikolopoulou, Shashikanth Ponnala, Caterina F. Ramogida, Andrew K. H. Robertson, Cristina Rodríguez-Rodríguez, Paul Schaffer, Clarence Williams, John W. Babich, Valery Radchenko, and Justin J. Wilson. An eighteen-membered macrocyclic ligand for actinium-225 targeted alpha therapy. *Angewandte Chemie International Edition*, 56(46):14712–14717, October 2017.

[3] Tonya Vitova, Ivan Pidchenko, David Fellhauer, Tim Pruessmann, Sebastian Bahl, Kathy Dardenne, Tadahiro Yokosawa, Bernd Schimmelpfennig, Marcus Altmaier, Melissa Denecke, Jörg Rothe, and Horst Geckeis. Exploring the electronic structure and speciation of aqueous and colloidal pu with high energy resolution xanes and computations. *Chemical Communications*, 54(91):12824–12827, 2018.

[4] J. J. Rehr, R. C. Albers, and S. I. Zabinsky. High-order multiple-scattering calculations of x-ray-absorption fine structure. *Physical Review Letters*, 69(23):3397–3400, December 1992.

[5] Bianca Schacherl, Michelangelo Tagliavini, Hanna Kaufmann-Heimeshoff, Jörg Göttlicher, Marinella Mazzanti, Karin Popa, Olaf Walter, Tim Pruessmann, Christian Vollmer, Aaron Beck, Ruwini S. K. Ekanayake, Jacob A. Branson, Thomas Neill, David Fellhauer, Cedric Reitz, Dieter Schild, Dominique Brager, Christopher Cahill, Cory Windorff, Thomas Sittel, Harry Ramanantoanina, Maurits W. Haverkort, and Tonya Vitova. Resonant inelastic x-ray scattering tools to count 5f electrons of actinides and probe bond covalency. *Nature Communications*, 16(1):1221, February 2025.

[6] Matthias Ernzerhof and Gustavo E. Scuseria. Assessment of the Perdew–Burke–Ernzerhof exchange-correlation functional. *The Journal of Chemical Physics*, 110(11):5029–5036, 03 1999.

[7] Harry Ramanantoanina. A dft-based theoretical model for the calculation of spectral profiles of lanthanide m4, 5-edge x-ray absorption. *The Journal of Chemical Physics*, 149(5):054104, August 2018.

[8] Pavel Zasimov, Lucia Amidani, Marius Retegan, Olaf Walter, Roberto Caciuffo, and Kristina O. Kvashnina. Herfd-xanes and rixs study on the electronic structure of trivalent lanthanides across a series of isostructural compounds. *Inorganic Chemistry*, 61(4):1817–1830, January 2022.
